# Can epigenetics shine a light on the biological pathways underlying major mental disorders?

**DOI:** 10.1017/S0033291721005559

**Published:** 2022-07

**Authors:** Luis Alameda, Giulia Trotta, Harriet Quigley, Victoria Rodriguez, Romayne Gadelrab, Daniella Dwir, Emma Dempster, Chloe C. Y. Wong, Marta Di Forti

**Affiliations:** 1 Department of Psychosis Studies, Institute of Psychiatry, Psychology and Neuroscience, King's College London, London, UK; 2 Departamento de Psiquiatría, Centro Investigación Biomedica en Red de Salud Mental (CIBERSAM), Instituto de Biomedicina de Sevilla (IBIS), Hospital Universitario Virgen del Rocío, Universidad de Sevilla, Sevilla, Spain; 3 Social, Genetic and Developmental Psychiatry Centre, Institute of Psychiatry, King's College London, London, UK; 4 Centre for Affective Disorders, Institute of Psychiatry, Psychology and Neuroscience, King's College London, London, UK; 5 Department of Psychiatry, Center for Psychiatric Neuroscience, Lausanne University Hospital (CHUV), Lausanne, Switzerland; 6 University of Exeter Medical School, University of Exeter, Barrack Road, Exeter, UK; 7 South London and Maudsley NHS Foundation Trust, London, UK

**Keywords:** Epigenetics, childhood trauma, DNA-methylation, mental health disorders

## Abstract

A significant proportion of the global burden of disease can be attributed to mental illness. Despite important advances in identifying risk factors for mental health conditions, the biological processing underlying causal pathways to disease onset remain poorly understood. This represents a limitation to implement effective prevention and the development of novel pharmacological treatments. Epigenetic mechanisms have emerged as mediators of environmental and genetic risk factors which might play a role in disease onset, including childhood adversity (CA) and cannabis use (CU). Particularly, human research exploring DNA methylation has provided new and promising insights into the role of biological pathways implicated in the aetio-pathogenesis of psychiatric conditions, including: monoaminergic (Serotonin and Dopamine), GABAergic, glutamatergic, neurogenesis, inflammatory and immune response and oxidative stress. While these epigenetic changes have been often studied as disease-specific, similarly to the investigation of environmental risk factors, they are often transdiagnostic. Therefore, we aim to review the existing literature on DNA methylation from human studies of psychiatric diseases (i) to identify epigenetic modifications mapping onto biological pathways either transdiagnostically or specifically related to psychiatric diseases such as Eating Disorders, Post-traumatic Stress Disorder, Bipolar and Psychotic Disorder, Depression, Autism Spectrum Disorder and Anxiety Disorder, and (ii) to investigate a convergence between some of these epigenetic modifications and the exposure to known risk factors for psychiatric disorders such as CA and CU, as well as to other epigenetic confounders in psychiatry research.

## Introduction of main epigenetic processes in psychiatry research

Both genetic and environmental factors are implicated in the aetiology of psychiatric disorders, however, the key causal mechanisms for guiding effective prevention and treatment remain poorly understood (Van Os, Rutten, & Poulton, 2008). Genetic association studies (Ripke et al., 2014) as well as epidemiological studies addressing the impact of the environment (van Os, Kenis, & Rutten, 2010) on disease burden, have not yet explained the non-complete genetic correlation between monozygotic twins in conditions such as schizophrenia (SCZ) (41–65%), Bipolar Disorder (BD) (~60%) (Craddock, O'Donovan, & Owen, 2005) or Major Depression (MDD) (~40%) (Ripke et al., 2013).

In the past decade, growing evidence has shown a link between epigenetic processes, and a range of mental health disorders (Binder, 2017). Epigenetic modifications refer to functional changes in DNA structural packaging or associated proteins without structural alteration of the DNA sequence itself (Jaenisch & Bird, 2003). This biological mechanism has important implications on how genes are expressed and how the chromatin is packaged, thus modifying subsequent protein translation within regionally specific parts of the central nervous system (Binder, 2017). The most studied epigenetic process in humans is DNA methylation (DNAm) (Table 1 for definitions of key terms). Indeed, recent parallel evidence suggests that differential DNAm profiles are associated with exposure to childhood adversity (CA) as well as cannabis use (CU) (Kandaswamy et al., 2020; Markunas et al., 2020; Nöthling, Malan-Müller, Abrahams, Hemmings, & Seedat, 2020). This suggests that epigenetic factors may account for some of the non-explained variance in genetics studies and possibly mediate the interactions between genotype and known environmental risk factors in influencing the onset of complex diseases (Relton & Smith, 2010).

**Table 1.** A glossary of key epigenetic terms and biological function of genes involved in pathways discussed in this review

Gene names and key terms	Biological function and definition
**Epigenetic terms**	
*DNA-methylation*	DNA-meth is the covalent addition of a methyl group to the 5^th^ carbon of a Cytosine (C) base, resulting in a 5-methylcytosine (5-mC) base. Epigenetic is the major process by which the environment can alter gene expression
*Candidate gene approach*	Explores methylation on certain genes of interests based on a priory hypothesis. It often examines whether DNAm changes in different CG sites within specific genes are related to a particular phenotype.
*Epigenome-Wide Association Studies*	Examines the association of DNAm changes (otherwise called methylome-wide association studies (MWAS)) across the entire genome for a particular phenotype, using a hypothesis-free paradigm. EWAS have been performed with increasingly powerful techniques and have moved from pioneer CpG-island microarrays studies that interrogated around 12.000 sites across the DNA (Mill et al., 2008) to more advanced techniques such as Infinium MethylationEPIC BeadChip, that covers more than 850 000 CpG methylation sites (Yong et al., 2016).
*Histone acetylation studies*	Histone acetylation is a dynamic epigenetic modification that functions in the regulation of DNA-templated reactions, such as transcription. This lysine modification is reversibly controlled by histone (lysine) acetyltransferases and deacetylases.
Methylome-wide association studies (MWAS)	Test a genome-wide set of methylation sites for association with an outcome of interest.
**Serotoninergic pathway**	
SLC6A4	Regulated serotonergic signalling via transporting 5-HT from synaptic spaces into presynaptic neurons. SLCA2 is involved in the recapture of the Norepinephrine
5-HTR (1A, 2A, 2B 3A, 5A)	These genes encode for the receptors for the neurotransmitter serotonin
A (MAOA)	One of two neighbouring gene family members that encode mitochondrial enzymes which catalyse the oxidative deamination of amines, such as dopamine, norepinephrine, and serotonin
**Dopaminergic pathway**	
DRD (2^a^, 3, 4)	Encode different subtypes of the dopamine receptor
COMT^a^(D1)	Encodes for Catechol-O-methyltransferase enzyme, which catalyses the transfer of a methyl group from S-adenosylmethionine to catecholamines, including the neurotransmitters dopamine, epinephrine, and norepinephrine important in the degradation of Dopamine (DA)
DAT1^a^	The dopamine transporter is implicated in a number of dopamine-related disorders, including attention deficit hyperactivity disorder, bipolar disorder, clinical depression, alcoholism, and substance use disorder
FAM63B	Involved in four networks regulated by miRNA, three of which are linked to neuronal differentiation and dopaminergic gene expression
SLC6A3	Provides instructions for making a protein called the dopamine transporter or DAT
**Glutamatergic/GABAergic pathway**	
GAD1	Encodes one of several forms of glutamic acid decarboxylase, an enzyme which is responsible for catalysing the production of gamma-aminobutyric acid from L-glutamic acid
PVALB	Encodes for Parvalbumin protein, essential for neural synchronisation in some neurons in the CNS
GRIN1 (2,2B 3B, D1)	The protein encoded by this gene is a critical subunit of N-methyl-D-aspartate receptors, members of the glutamate receptor channel superfamily. It plays an important role in the plasticity of synapses
GRIA 2, 3	Encodes for the Glutamate Ionotropic Receptor AMPA Type Subunit 2 and 3 Glutamate receptors, which are the predominant excitatory neurotransmitter receptors in the mammalian brain
MARLIN-1	(synonym of JAKMIP1) codes for a protein that may play a role in the microtubule-dependent transport of the GABA-B receptor
KCNJ6	Encodes a member of the G protein-coupled inwardly-rectifying potassium channel family of inward rectifier potassium channels. This type of potassium channel allows a greater flow of potassium into the cell than out of it and thus regulates circuit activities in neural cells. Expressed in GABAergic synapses
HELT	Protein Coding gene involved in *DNA-binding transcription factor activity* and *protein dimerisation activity*. It is a transcriptional repressor gene which is known to function as a selector gene that determines GABAergic over glutamatergic fate in the mesencephalon
GRIK2	Codes for the Glutamate Ionotropic Receptor Kainate Type Subunit 2. Glutamate receptors are the predominant excitatory neurotransmitter receptors in the mammalian brain
SLC6A12	Transports betaine and GABA. May have a role in the regulation of GABAergic transmission in the brain through the reuptake of GABA into presynaptic terminals, as well as in osmotic regulation.
GABBR1, 2	Encodes a receptor for GABA that functions as a heterodimer with GABA(B) receptor 1 and 2. Defects in this gene may underlie brain disorders such as schizophrenia and epilepsy.
GRIN3B	The protein encoded by this gene is a subunit of an N-methyl-D-aspartate (NMDA) receptor. The encoded protein is found primarily in motor neurons, where it forms a heterotetramer with GRIN1 to create an excitatory glycine receptor. Variations in this gene have been proposed to be linked to schizophrenia
**Neurogenesis**	
RELN	This gene encodes a large secreted extracellular matrix protein (Reelin) thought to control cell-cell interactions critical for cell positioning and neuronal migration during brain development. expressed in GABAergic interneurons
BDNF	Encodes the brain-derived neurotrophic factor (BDNF), a protein involved in promoting the survival, growth and differentiation of new neurons and synapses
POU5F1, POU6F2. POU3F1	Encodes a transcription factor protein that binds to the octamer motif (5-ATTTGCAT-3) and controls myelination (thought to be involved in embryogenesis and neurogenesis)
NPDC1	Encored for a protein that Suppresses oncogenic transformation in neural and non-neural cells and down-regulates neural cell proliferation. Might be involved in transcriptional regulation
PI3K	Phosphatidylinositol 3-kinases, are a family of enzymes involved in cellular functions such as cell growth, proliferation, differentiation, motility, survival and intracellular trafficking
CUX1^a^	Encodes a member of the homeodomain family of DNA binding proteins that regulates gene expression, morphogenesis, and differentiation and it also plays a role in cell cycle progression
CLMN^a^	Encodes calmin (calponin-like transmembrane domain protein)
SENP7^a^	Encodes sentrin-specific protease 7
**Immune system and inflammation**	
ZC3H12D	It is a Protein (Zinc Finger CCCH-Type Containing 12D) Coding gene, which in association with ZC3H12A enhances the degradation of interleukin IL-6 mRNA level in activated macrophages, among other functions
TCF3	This gene encodes a member of the E protein (class I) family of helix-loop-helix transcription factors. E proteins play a critical role in lymphopoiesis, and the encoded protein is required for B and T lymphocyte development, among other functions
IKZF4	Members of the Ikaros family of transcription factors, which includes Eos, are expressed in lymphocytes and are implicated in the control of lymphoid development
YOD1	Protein ubiquitination controls many intracellular processes, including cell cycle progression, transcriptional activation, and signal transduction, involved in IL-1 signalling to NF-*κ*B
IL17RA	Code for Interleukin 17A (IL17A), which is a proinflammatory cytokine secreted by activated T-lymphocytes
TLR1 (3)	Encodes Toll-Like Receptor 1, family which plays a fundamental role in pathogen recognition and activation of innate immunity
TNFRSF13C	TNF Receptor Superfamily Member 13C, a membrane protein of the TNF receptor superfamily which recognises BAFF, an essential factor for B cell maturation and survival
HERC5	This gene is a member of the HERC family of ubiquitin ligases and encodes a protein with a HECT domain and five RCC1 repeats. Pro-inflammatory cytokines upregulate expression of this gene in endothelial cells
FCGR2B	One of the genes thought to influence susceptibility to several autoimmune diseases in humans inhibiting the functions of activating Fc*γ*Rs, such as phagocytosis and pro-inflammatory cytokine release
PIK3R3	Plays an important role in the regulation of cellular lipid metabolism
INPP5D	Encodes Src homology 2 (SH2) domain-containing inositol polyphosphate 5-phosphatase 1 (SHIP1) that functions as a negative regulator of cell proliferation and survival
FCGR2C, 2B	Encodes one of three members of a family of low-affinity immunoglobulin gamma Fc receptors found on the surface of many immune response cells and involved in phagocytosis
IGHA1	Encodes a constant (C) segment of Immunoglobulin A heavy chain that plays a critical role in immune function in the mucous membranes
FCAR	Codes for the transmembrane receptor Fc*α*RI, also known as CD89 (Cluster of Differentiation 89), that plays a role in both pro- and anti-inflammatory responses
CD224	This gene is a human gamma-glutamyltransferase catalyses the transfer of the glutamyl moiety of glutathione to a variety of amino acids and dipeptide acceptors
LAX1	A membrane-associated adaptor protein mainly expressed in B cells, T cells, and other lymphoid-specific cell types
TXK	A member of Tec family nonreceptor tyrosine kinase, is expressed on Th1/Th0 cells, and Txk regulates specifically IFN-gamma gene expression
PRF1	Encodes perforin a pore-forming cytolytic protein found in the granules of cytotoxic T lymphocytes (CTLs) and natural killer cells (NK cells)
CD7	Encodes a transmembrane protein which is a member of the immunoglobulin superfamily found on thymocytes and mature T cells that plays an essential role in T-cell interactions and also in T-cell/B-cell interaction during early lymphoid development
MPG	Encodes N-methylpurine DNA glycosylase a specific type of DNA glycosylase involved in the recognition of a variety of base lesions, including alkylated and deaminated purines, and initiating their repair via the base excision repair pathway
MPOG	A member of the XPO subfamily of peroxidase enzyme most abundantly expressed in neutrophil granulocytes
MARC2^a^	The protein encoded by this gene is an enzyme found in the outer mitochondrial membrane that reduces N-hydroxylated substrates
CEMIP^a^	Cell migration-inducing and hyaluronal-binding protein, known as KIAA1199, has been shown to bind hyaluronic acid and catalyse its depolymerisation its depolymerisation independently of CD44 and hyaluronidases
**Oxidative stress**	
GGT6	Encored for a gamma-glutamyltransferase, that plays a key role in glutathione homoeostasis by providing substrates for its synthesis
GSTM5	(Glutathione S-Transferase Mu 5), important for glutathione homoeostasis
**Hypothalamus pituitary adrenal axis pathway**	
NR3C1	Encodes the human glucocorticoid receptor protein, which is the receptor to which cortisol and other glucocorticoids bind
miR124	A microRNA that targets NR3C1
FKBP5 (2, 1B)	Encodes the FK506 binding protein, a member of the immunophilin protein family which may play a role in immunoregulation and basic cellular processes involving protein folding and trafficking
SKA2	Encodes for a component of the spindle and kinetochore-associated protein complex, which is a protein complex involved in regulating chromosomal segregation. SKA2 is important in facilitating GR nuclear transactivation.
**Cannabinoid system**	
CNR1 and CNR2^a^	Encodes the cannabinoid receptor gene
**Other genes**	
DNMTS	This gene encodes an enzyme that transfers methyl groups to cytosine nucleotides of genomic DNA
OXTR	Encodes oxytocin, which is a neuropeptide hormone produced by the hypothalamus and released into systemic circulation by the posterior pituitary
**Tobacco signature genes**	
AHRR^a^	The protein encoded by this gene participates in the aryl hydrocarbon receptor (AhR) signalling cascade, which mediates dioxin toxicity, and is involved in the regulation of cell growth and differentiation
F2RL3^a^	Encodes a member of the protease-activated receptor subfamily, part of the G-protein coupled receptor 1 family of proteins. This receptor plays a role in blood coagulation, inflammation and response to pain
GFI1	Encodes a nuclear zinc finger protein that functions as a transcriptional repressor. This protein plays a role in diverse developmental contexts, including haematopoiesis and oncogenesis
MYO1G	Is a plasma membrane-associated class I myosin that is abundant in T and B lymphocytes and mast cells.. This myosin is required during immune response for detection of rare antigen-presenting cells by regulating T-cell migration

a genes related to cannabis use.

Initially, epigenetic research in psychiatry used a candidate gene approach, and progressively, research moved to Epigenome Wide Association studies (EWAS) (Table 1). While both designs have their advantages and limitations, the breadth of coverage of EWAS offers a more informative insight on biological pathways. This is based on the rational that chromatin conformation and transcriptional regulation are influenced by a set of methylated or unmethylated cytosines across a region, rather than specific CpG sites in isolation (Mill et al., 2008)

Different biological pathways have been implicated in the aetio-pathogenesis across multiple mental disorders. Some of these are pathways related to neurotransmission such as serotonin (Provenzi, Giorda, Beri, & Montirosso, 2016), dopamine or GABA/glutamatergic processes (McCutcheon, Krystal, & Howes, 2020); while others pathways involve inflammation (Cullen et al., 2019), oxidative stress (Steullet et al., 2016), synaptic plasticity and neurogenesis (Claudino, Gonçalves, Schuch, Martins, & Rocha, 2020), or the stress response system (Hypothalamic Pituitary adrenal Axis – HPA) (Wesarg, Van Den Akker, Oei, Hoeve, & Wiers, 2020). It is important to take into account that some of these processes participate in disease pathogenesis in a parallel manner, such as via the redox system and through the glutamatergic/GABAergic imbalance (Hardingham & Do, 2016); or the immune system and the stress response (Pariante, 2017). Although these processes are often explored within discrete categorical clinical conditions, they often overlap transdiagnostically. For instance, alterations in serotonin pathways are linked to both depression and psychosis phenotypes (Selvaraj, Arnone, Cappai, & Howes, 2014).

In this review, we set to appraise firstly, the evidence of DNAm modifications both from candidate genes and EWAS studies, associated either specifically or transdiagnostically with psychiatric conditions, and secondly, if these DNAm modifications map onto biological pathways. Thirdly, we will explore if the existing findings from studies on DNAm changes associated with CA and CU, two of the environmental exposures most consistently associated with psychiatric disorders (Lindert et al., 2014; Mandelli, Petrelli, & Serretti, 2015; Marconi, Di Forti, Lewis, Murray, & Vassos, 2016; Sideli, Quigley, La Cascia, & Murray, 2020a; Varese et al., 2012), point at the same biological pathways therefore contributing to the understanding of how these environmental exposures increase transdiagnostic and specific psychiatric liability. Details on methodological considerations can be found in Online Supplementary Material (SM).

## Evidence of epigenetic processes in major transdiagnostic pathways

**Fig. 1.**
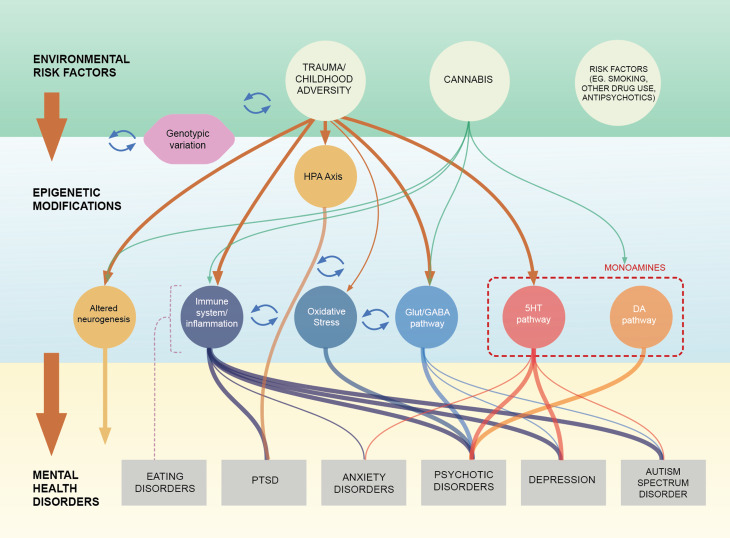
Summary of the evidence on potential pathways linking childhood trauma and cannabis use with psychiatric conditions through DNAm changes. *Note:* This figure summarises the evidence presented in this review, highlighting the idea that some biological pathways linking environmental risk factors with mental health disorders via epigenetic changed in the form of DNAm are transdiagnostics (e.g immune system/inflammation) while others seem to be more specific (e.g dopaminergic system). (1) The environmental risk factors row and epigenetic modifications row suggest links between childhood adversity (CA), and Cannabis use (CU) and DNAm changes mapping to biological pathways which are also functionally related (Serotoninergic, Dopaminergic pathways, Glutamatergic & GABAergic pathway, Neurogenesis, Immune system & Inflammation and Oxidative stress). (2)The epigenetic modifications row and mental health disorders row illustrate the evidence, from case–control studies, of an association between DNAm changes in these pathways and the major mental health conditions (Eating Disorders (anorexia nervosa and bulimia nervosa) Post-traumatic stress disorder, Anxiety Disorders, Psychotic Disorder, Bipolar disorders, Depression and Autism Spectrum Disorders). (3) The arrows connecting the three rows show the potential mediating role of DNAm changes linking CA and CU and risk to develop mental health conditions. The thickness of the lines shows the robustness of the evidence reported in the literature review. The items “genotype: and “other risk factors” are added to highlight the influence of genetic factors and environmental confounders in DNAm studies. The dotted line connecting eating disorders with the pathways indicate that literature was limited and mixed not allowing to draw clear links with the pathways.

**Fig. 2.**
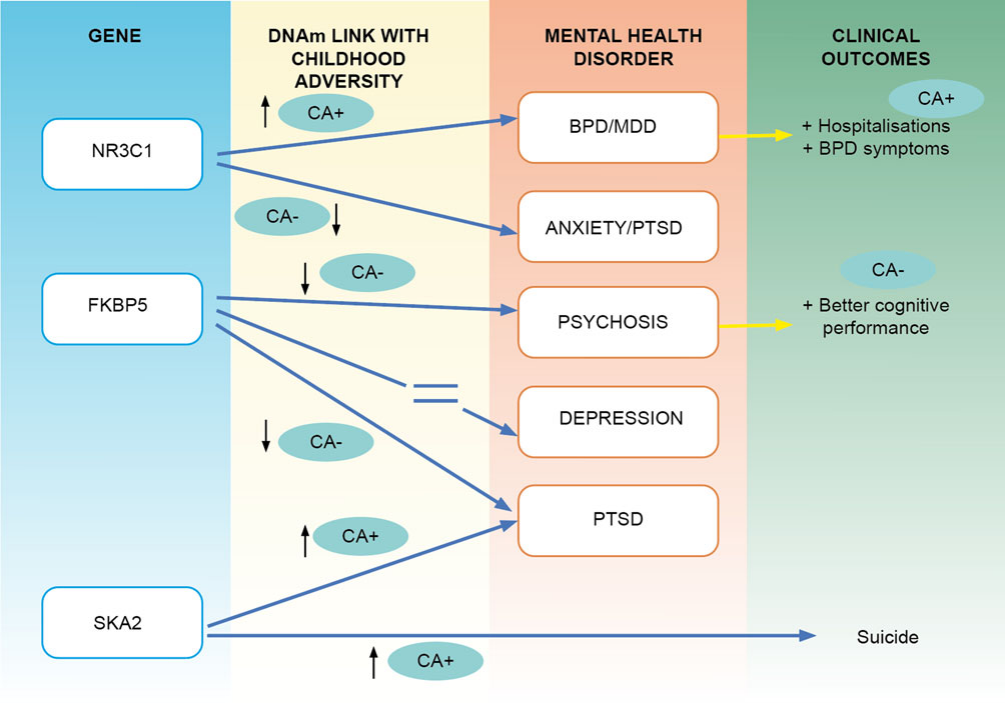
Summary of the evidence linking childhood adversity and DNAm changes on the Hipotalamic Pituitary Adrenal Axis in various conditions as well as with some clinical measures. *Note:* This Figure illustrates the evidence from candidate gene studies linking childhood adversity (CA) with DNAm in CpG sites located in *NR3C1, FKBP5, SKA2* and CA, with various conditions and various clinical outcomes. In the gene and DNAm columns, CA + (with an arrow pointing up) reflects the presence of a positive association between the DNAm in probes located in those genes and CA; CA- (with an arrow pointing down) reflects a negative association. The disorder column shows in which mental health condition that association has been found. Lastly, the clinical outcomes column shows the presence of evidence linking DNAm, with a particular clinical phenotype; CA + indicated that the association between DNAm and the clinical outcome was related to CA.

In this section we will review the evidence, predominantly from case–control studies pointing at an association between DNAm changes in the Serotoninergic, Dopaminergic pathways, Excitatory inhibitory balance (including the Glutamatergic and GABAergic dysfunction), Synaptic plasticity and Neurogenesis; the Immune system, Inflammation and Oxidative stress and the major mental conditions (focusing on Eating Disorders (ED): anorexia nervosa (AN) and bulimia nervosa (BN), Autism Spectrum Disorder (ASD), BD and Psychotic Disorder, Depression, Post Traumatic Stress Disorder (PTSD) and Anxiety Disorders). Summary of findings is illustrated in Fig. 1; findings on HPA-axis and its association to environmental risk factors are presented in Section ‘The epigenetic signature of childhood adversity and cannabis use’ and Fig. 2 and Online Supplementary Table S1 (SM) summarises the characteristics of the articles mentioned in that section. Table 2 summarised the key elements of studies finding evidence of a link between DNAm on genes involved in each biological pathway across all disorders. Screening

**Table 2.** Summary of the direction of the associations between DNAm, mental health disorders and clinical or biological outcomes presented in this review

Gene (hyper ↑ or ↓ hypo DNAm) and citation	Candidate gene or EWAS	Tissue	Condition/sample	Clinical/biological outcome
**Serotoninergic pathway**				
SLC6A4				
↑Abdolmaleky et al. (2014)	Candidate gene	PMB/saliva	SCZ	
Kang et al. (2013)	Candidate gene	Blood	Depression	CA → ↑SLC6A4 → clinical severity
Olsson et al. (2010)	Candidate gene	Buccal	Depression	↑SLC6A4 → ↑ Depressive symptoms
↑Philibert et al. (2008)	Candidate gene	Blood	Depression	↑SLC6A4 → ↑ history MDD
↑Zhao et al. (2013)	Candidate gene	Blood	Twin male veterans	↑SLC6A4 → ↑ Depressive symptoms
Perez-Cornago et al. (2014)	Candidate gene	Blood	General population	↑SLC6A4 → decrease depressive symptoms from baseline to Follow-up
Boehm et al. (2019)	Candidate gene	Blood	Anorexia nervosa	↑SLC6A4 → resting-state functional connectivity→ anorexia symptoms
Koenen et al. (2011)	Candidate gene	Blood	PTSD	CA + ↓SLC6A4 → PTSD
Peng et al. (2018)	Candidate gene	Blood	General population	CA → ↑SLC6A4 → depressive symptoms
Schiele et al. (2019)	Candidate gene	Blood	Panic disorder	↑SLC6A4→ Comorbid depression
5-HTR 1A				
↑Carrard et al. (2011)	Candidate gene	Blood	SCZ/BD	
5-HTR 2A				
↑Cheah et al. (2017)	Candidate gene	PMB	SCZ	
↑Abdolmaleky et al. (2011)	Candidate gene	PMB	SCZ/BD	
↑Hranilovic et al. (2016)	Candidate gene	Blood	ASD	
5-HT3A-R				
↑Perroud et al. (2016)	Candidate gene	Blood	BD/Borderline PD	CA → ↑*5-HT3A-R* → clinical severity
A MAOA				
↓ Ziegler et al. (2016)	Candidate gene	Blood	Panic disorder	↑ → Better response to CBT in agoraphobic symptpms
↓Schiele et al. (2018)	Candidate gene	Blood	Agoraphobia	
↓Domschke et al. (2012)	Candidate gene	Blood	Panic disorder	CA → ↓*A* (*MAOA*)
Peng et al. (2018)	Candidate gene	Blood	General population	CA → ↓*A* (*MAOA*) → depressive symptoms
**Dopaminergic pathway**				
DRD2				
↑Kordi-Tamandani et al. (2013b)	Candidate gene	Blood	SCZ	
↓Yoshino et al. (2016)	Candidate gene	Blood	SCZ	
↑Frieling et al. (2010)	Candidate gene	Blood	Anorexia and bulimia nervosa	
DRD3				
↑Dai et al. (2014)	Candidate gene	Blood	SCZ	
DRD4				
↑Cheng et al. (2014)	Candidate gene	Blood	SCZ	
↑Kordi-Tamandani et al. (2013b)	Candidate gene	Blood	SCZ	
DRD5				
↑Kordi-Tamandani et al. (2013b)	Candidate gene	Blood	SCZ	
MB-COMT				
↓Abdolmaleky et al. (2006)	Candidate gene	PMB	SCZ/BD	
↓Nohesara et al. (2011)	Candidate gene	Saliva	SCZ/BD	
Walton et al. (2014)	Candidate gene	Blood	SCZ	↑ *MB-COMT* ->better neural activity in left DLPFC
↓Nour El Huda et al. (2018)	Candidate gene	Blood	SCZ	↑ *MB-COMT->* ↓excited and depressed symptoms
S-COMT				
↑Murphy et al. (2005)	Candidate gene	PMB	SCZ	
↑Melas et al. (2012)	Candidate gene	Blood	SCZ	
COMTD1				
↓Nishioka et al. (2013)	EWAS	Blood	SCZ	
SLC6A3				
↓Nishioka et al. (2013)	EWAS	Blood	SCZ	
DAT1*				
↑Frieling et al. (2010)	Candidate gene	Blood	Anorexia and bulimia nervosa	
FAM63B				
↓Aberg et al. (2012)	EWAS	Blood	SCZ	
**Glutamatergic/GABAergic pathway (Excitatory/inhibitory balance)**				
PVALB				
↑Fachim et al. (2018)	Candidate gene	PMB	SCZ	
GMR2, GMR5				
↓Kordi-Tamandani et al. (2013a)	Candidate gene	Blood	SCZ	
GRIA 3				
↑Kordi-Tamandani et al. (2013a)	Candidate gene	Blood	SCZ	
GRIA 2				
↓Mill et al. (2008)	EWAS	PMB	SCZ/BD	
↓Aberg et al. (2012)	EWAS	Blood	SCZ	
GRIA 4				
↑Numata et al. (2014)	EWAS	PMB	SCZ	
GABBR1				
↑Hannon et al. (2021)	EWAS	Blood	Psychosis and SCZ	
GABBR2				
↑Pun et al. (2011)	Candidate gene	Blood	SCZ	
↑Zong et al. (2017)	Candidate gene	Blood	SCZ	
GRIN 2B				
↓Fachim et al. (2019)	Candidate gene	Blood	SCZ	CA → ↑*GRIN2B*
Engdahl et al. (2021)	Candidate gene	Saliva	General population	
GRIND1				
Weder et al. (2014)	EWAS	Saliva	Trauma/non-trauma children	
GAD1				
Ruzicka et al. (2015)	Candidate gene	PMB	SCZ/BD	
↓Domschke et al. (2013)	Candidate gene	Blood	Panic disorder	Life events → ↓GAD1 DNAm
GRIN3B				
↓Mill et al. (2008)	EWAS	PMB	SCZ/BD	
MARLIN-1				
↑Mill et al. (2008)	EWAS	PMB	SCZ/BD	
KCNJ6				
↑Mill et al. (2008)	EWAS	PMB	SCZ/BD	
HELT				
↑Mill et al. (2008)	EWAS	PMB	SCZ/BD	
GRIK2				
Nagy et al. (2015)	EWAS	PMB	Depression	
SLC6A12				
↑Hannon et al. (2021)	EWAS	Blood	Psychosis and SCZ	
**Synaptic plasticity and neurogenesis**				
RELN				
Tamura et al. (2007)	Candidate gene	PMB	SCZ/BD	↓DNAm → poor cognition
Alfimova et al. (2018)	Candidate gene	Blood	SCZ	
Fikri et al. (2017)	Candidate gene	Blood	SCZ	
PI3K				
Wong et al. (2019)	EWAS	PMB	ASD	
BDNF				
↑Ursini et al. (2016)	Candidate gene	Blood	SCZ	
↑Duffy et al. (2019)	Candidate gene	Saliva	BD	
↑Dell et al. (2014)	Candidate gene	Blood	Unipolar, BD and MDD	
↑Kim et al. (2017)	Candidate gene	Blood	PTSD	
↑Kang et al. (2015)*	Candidate gene	Blood	Depression	↑BDNF->↑depressive symptoms
↑Peng et al. (2018)	Candidate gene	Blood	Depression	CA -> ↑BDNF→ depressive symptoms
↑Thomas et al. (2018)	Candidate gene	Saliva	Borderline PD	
D'Addario et al. (2019)	Candidate gene	Blood	OCD	
↑Thaler et al. (2014)	Candidate gene	Blood	Bulimia nervosa	CA + Borderline PD →↑BDNF meth
Moser et al. (2015)	EWAS	Saliva	PTSD	CA → ↑BDNF meth / ↑BDNF meth-> maternal anxiety
Weder et al. (2014)	EWAS	Saliva/blood	Trauma/non-trauma children	Differently methylated between CA + and CA-
POU6F2				
Comes et al. (2020)	EWAS	Blood	BD	↑CA-> ↓POU6F2
POU5F1				
Arranz et al. (2021)	EWAS	Blood	Borderline PD	↑CA-> ↓POU5F1
POU3F1				
Lutz et al. (2017)	EWAS	PMB	Depression	↑CA-> ↓POU3F1
CUX1*				
Osborne et al. (2020)	EWAS	Blood	General population	Differently methylated in CU (exploratory analyses)
CLMN*, SENP7*				
Clark et al. (2021)	EWAS	Blood	Adolescents	Differently methylated in CU users
**Immune system and inflammation**				
ZC3H12D				
↓Montano et al. (2016)	EWAS	Blood	SCZ	
TCF3				
↑Montano et al. (2016)	EWAS	Blood	SCZ	
IKZF4				
↓Montano et al. (2016)	EWAS	Blood	SCZ	
YOD1				
↑Hüls et al. (2020)	EWAS	PMB	Depression	
IL17RA				
Prados et al. (2015)	EWAS	Blood	Borderline PD/depression	CA → ↑ IL17RA
TLR1 3				
Uddin et al. (2010)	EWAS	Blood	PTSD	CA → ↓TLR1/3
TNFRSF13C				
Arranz et al. (2021)	EWAS	Blood	Borderline PD	Differently methylated in CA exposed
*FCGR2B, PIK3R3, INPP5D, INPP5D, IGHA1, FCAR*	EWAS	Blood	SCZ	
Aberg et al. (2014)				
*CD224, LAX1, TXK, PRF1, CD7, MPG, MPOG*	EWAS	Blood	SCZ	
Liu et al. (2014)				
MARC2*				
Osborne et al. (2020)	EWAS	Blood	General population	Differently methylated in CU and tobacco users
CEMIP*	EWAS	Blood	General population	Differently methylated in CU
Markunas et al. (2020)				
Oxidative stress				
↑GSTM5				
Kebir et al. (2017)	EWAS	Blood	At the risk of psychosis	↑*GSTM5* in converters *v.* non-converters
GGT6				
Arranz et al. (2021)	EWAS	Blood	Borderline PD	
**Hypotalamus pituitary-adrenal axis pathway**				
NR3C1				
Bustamante et al. (2016)	Candidate gene	Blood	Depression	CA → ↓ *NR3C1* DNAm
Farrell et al. (2018)	Candidate gene	Blood	Depression	CA → ↑ *NR3C1* DNAm
Martin-Blanco et al. (2014)	Candidate gene	Blood	Borderline PD	CA → ↑ *NR3C1* DNAm→ clinical severity
Perroud et al. (2011)	Candidate gene	Blood	Borderline PD /MDD	CA→ ↑ *NR3C1* DNAm
Radtke et al. (2015)	Candidate gene	Blood	General population	CA + ↑ *NR3C1* DNAm → Borderline PD symptoms
Labonte et al. (2014)	Candidate gene	Blood	PTSD	PTSD + → ↓ *NR3C1* DNAm
Schechter et al. (2015)	Candidate gene	Saliva	PTSD	PTSD + → ↓ *NR3C1* DNAm
Yehuda et al. (2015)	Candidate gene	Blood	PTSD	PTSD + → ↓ *NR3C1* DNAm
↑ Wang et al. (2017)	Candidate gene	Blood	GAD	CA → ↓ *NR3C1* DNAm
Peng et al. (2018)	Candidate gene	Blood	General population	CA → ↑ *NR3C1*→ depressive symptoms
FKBP5				
Tozzi et al. (2018)	Candidate gene	Blood	Depression	CA → ↓ *FKBP5* DNAm
Misiak et al. (2020)	Candidate gene	Blood	SCZ	CA → ↓ *FKBP5* DNAm
Klengel et al. (2013)	Candidate gene	Blood	PTSD	CA → ↓ *FKBP5* DNAm
SKA2				
Kaminsky et al. (2015)	Candidate gene	Blood/saliva	General population	CA ↑ x SKA2↑-> suicide attempt
↑Sadeh et al. (2016a, 2016b)	Candidate gene	Blood	PTSD	CA↑-> SKA2↑-> cortical thickness

*extensive reviews cover the role of BDNF Methylation in depression (Hing et al., 2018), Schizophrenia (Di Carlo et al., 2019), and eating disorders (Thaler and Steiger, 2017), therefore studies mentioned here are just examples of the literature in this particular domain. When various genes are reported in the same pathway and the same study, but no specific information on clinical/biological outcome or specific direction if the association is provided, these genes have been put in the same row (e.g Asberg et al., and Liu et al.,). When an arrow is next to the author's name it reflects the direction of the DNAm of the particular gene in in relation to the condition ↑ : increased ↓: decreased DNAm. When in column 1 there is no arrow is because information could not be obtained or was not clear, and the presence of that gene indicates the association of DNAm in that gene with the respective condition (differently methylated). When a three step sequence separated by an arrow is presented, this refers to mediation analyses (e.g peng et al.,: CA → ↑SLC6A4 → depressive symptoms: DNAm of SLC6A mediates the effect of CA on depressive symptoms). CA: childhood adversity; CU: cannabis use. Definition of each gene is presented in Table 1. DLPFC: Dorsolateral prefrontal cortex. ASD: autism spectrum disorder; SCZ: schizophrenia. PTSD: post-traumatic stress disorder; Borderline PD: Borderline personality disorder; MDD: major depression disorder; BD: bipolar disorder.

### The serotoninergic pathway

There are preclinical and human studies pointing at an implication of the serotonin (5HT) system dysfunction in a broad range of psychiatric diseases (Kaye, Fudge, & Paulus, 2009). The strongest evidence is at the level of the serotonin transported genes (mainly *SLC6A4*) with candidate genes studies suggesting an increased in methylation in depression (Kang et al., 2013; Philibert et al., 2008; Zhao, Goldberg, Bremner, & Vaccarino, 2013), BD (Sugawara et al., 2011) and reporting a positive association with symptoms severity (Olsson et al., 2010), comorbid depression in those with panic disorder (Schiele et al., 2019), and improvement from baseline to follow-up (Perez-Cornago, Mansego, Zulet, & Martinez, 2014). It has been suggested that an increased DNAm of *SLC6A4* could repress gene expression, leading to decreased serotonin uptake and lower activity, which ultimately would lead to the manifestation of depressive symptoms (Chen, Meng, Pei, Zheng, & Leng, 2017)

A pattern of hypermethylation has also been found in samples of SCZ (Abdolmaleky et al., 2014). although with mixed evidence(Alelú-Paz et al., 2015). Candidate gene studies in SCZ and BD across various tissues (Abdolmaleky et al., 2011; Carrard, Salzmann, Malafosse, & Karege, 2011; Cheah, Lawford, Young, Morris, & Voisey, 2017) show elevated DNAm of the *5-HTR1A* and *5-HTR2A* genes respectively. Further, EWAS studies have identified differential DNAm in *HTR2A* (Numata, Ye, Herman, & Lipska, 2014), *HTR5A* and *HTR1E* (Nishioka et al., 2013; Pidsley et al., 2014) genes in those with psychosis.

Evidence on ED so far has not found an association with SLC6A4 DNAm and AN (Boehm et al., 2019; Pjetri et al., 2013; Steiger et al., 2019).

In ASD, preliminary evidence indicated higher *HTR2A* promoter DNAm in leucocytes of those carrying the high-risk genotype in the *HTR2A* (Hranilovic, Blazevic, Stefulj, & Zill, 2016).

Another well-explored gene of interest in the serotoninergic pathway is *MAO-A* (Shih & Thompson, 1999) which is involved in monoamine degradation and it has established linked with depression (Meyer et al., 2006). While studies in depression have found inconsistent DNAm changes (Domschke et al., 2015; Melas & Forsell, 2015; Melas et al., 2013); in candidate gene studies in anxiety disorders, the evidence points at a pattern of hypomethylation (Ziegler et al., 2016) as shown in acrophobia (Schiele et al., 2018) and obsessive compulsive disorder (OCD) (Domschke et al., 2012). Moreover, increased *MAO-A* DNAm has been suggested as a potential useful marker of better response to psychotherapy in anxiety disorders (Schiele et al., 2020; Ziegler et al., 2016).

Overall, we find a transdiagnostic link between DNAm changes in genes involved in the serotoninergic pathway, with limited evidence in ED (findings on PTSD discussed in Section ‘The epigenetic signature of childhood adversity and cannabis use’ and described in Table 2).

### The dopaminergic pathway

It is widely accepted that dopaminergic dysregulation stands as one of the most supported hypotheses for the pathogenesis of SCZ and psychosis as a whole (Jauhar et al., 2018; McCutcheon et al., 2020). Studies examining DNAm in the blood of patients with SCZ as compared with controls have showed both higher and lower DNAm levels in different DA receptor's genes; with decreased DNAm in *DRD3* (Dai et al., 2014) and *DRD4* (Cheng et al., 2014); and in other dopamine receptors (Kordi-Tamandani, Sahranavard, & Torkamanzehi, 2013b; Yoshino et al., 2016).

Hypomethylation of the *COMT* membrane-bound isoform, has been identified in samples of people with SCZ across tissues (Abdolmaleky et al., 2006; Nohesara et al., 2011; Nour El Huda et al., 2018; Walton et al., 2014), while the soluble isoform (*S-COMT*) has been reported to be hypermethylated (Melas et al., 2012; Murphy, O'Reilly, & Singh, 2005). EWAS studies comparing SCZ patients with controls have found hypomethylation of *SLC6A3*, a dopamine transporter (Nishioka et al., 2013), *COMTD1* and *FAM63B*, a gene linked to dopaminergic gene expression (Aberg et al., 2014).

In ED, findings of DNAm changes affecting dopaminergic genes *DAT* and *DRD2* are mixed (Frieling et al., 2010; Pjetri et al., 2013). It has been suggested that DNAm variation in the dopamine pathway in ED may be related to comorbid Borderline Personality Disorder (Borderline PD) (Groleau et al., 2014) and exposure to CA (Section ‘The epigenetic signature of childhood adversity and cannabis use’ and Online Supplementary Table S1 (SM)).

None of the EWAS studies conducted in ASD has found evidence supporting an association with DNAm changes involved in the Dopaminergic pathway.

Overall, recent findings support a link between DNAm changes in genes involved in the dopaminergic pathway related to neurodevelopmental disorders such as SCZ, with limited evidence suggesting a link with other conditions.

### Glutamatergic/GABAergic Pathway and excitatory/inhibitory balance

Alterations in glutamatergic and GABAergic pathways, which can lead to either excitatory/inhibitory imbalance, have been reported to play a role in the etiopathogenesis of psychotic disorders (McCutcheon et al., 2020) and ASD (Marotta et al., 2020). Furthermore, N-Methyl-D-aspartic acid or N-Methyl-D-aspartate (NMDAR) hypofunction as well as a decrease in the parvalbumin-expressing fast-spiking interneurons (PVI), both processes being essential for the excitatory/inhibitory balance, have been widely shown to be involved in psychotic disorders (Thuné, Recasens, & Uhlhaas, 2016).

In SCZ and psychosis, there is evidence from candidate genes studies across tissues supporting DNAm differences between cases and controls in genes such as the Parvalbumin (*PVALB*) gene(Fachim, Srisawat, Dalton, & Reynolds, 2018), *GMR2* and *GMR5* of the glutamatergic receptors (Kordi-Tamandani, Dahmardeh, & Torkamanzehi, 2013a); various CpG sites in the *β*2 subunit of the GABAa receptor gene (*GABRB2*) (Pun et al., 2011; Zong et al., 2017), and in *GRIN2B*, involved in the function of NMDAR (Fachim et al., 2019). A dysregulation of multiple DNAm positions in the regulatory network of *GAD1*, was identified in patients with SCZ and BD compared to controls (Ruzicka, Subburaju, & Benes, 2015).

In terms of EWAS Mill and colleagues (Mill et al., 2008) performed the first EWAS in post-mortem brains of SCZ and BD subjects compared to controls, and found DNAm changes associated with SCZ and BD at loci involved in glutamatergic (*GRIA 2, GRIND3B*) and GABAergic (*MARLIN-1, KCNJ6, HELT*) neurotransmission, supporting previous candidate genes results. Findings related to *GRIA* family genes have been replicated in latter EWAS studies (Aberg et al., 2012; Numata et al., 2014), and other EWAS studies have confirmed DNAm changes in genes involving GABAergic neurotransmission (*SLC6A12* and *GABBR1*) (Hannon et al., 2021).

In ASD, an EWAS study on histone acetylation in participants with the disorder compared to controls found an enrichment of hyperacetylated sites in genes involved in GABA receptor activity (Ramaswami et al., 2020), although this has not been previously found on DNAm (Wong et al., 2018).

Lastly, a Depression EWAS (Nagy et al., 2015) of post-mortem brains of suicide victims and controls found 115 differentially methylated regions (DMRs), which included regions related to *GRIK2*.

Overall, there is evidence linking DNAm changes on genes involved in the glutamatergic pathway mainly with psychosis, with some evidence suggesting a link with ASD and MDD

### Synaptic plasticity and neurogenesis

Synaptic plasticity anomalies are associated with psychiatric conditions and may account for various symptoms, such as cognitive deficits (Claudino et al., 2020; Di Carlo, Punzi, & Ursini, 2019; Lin & Huang, 2020).

*RELN* is a good studies gene that has been linked to SCZ (Costa et al., 2002). An aberrant DNAm status in *RELN* has been found in SCZ and BD patient as compared with controls (Fikri et al., 2017; Tamura, Kunugi, Ohashi, & Hohjoh, 2007). Interestingly, peripheral blood hypomethylation in the *RELN* promoter was associated with poor cognitive functioning (Alfimova, Kondratiev, Golov, & Golimbet, 2018).

In ASD, an EWAS study in post-mortem brain found dysregulation in the pathway of phosphatidylinositol 3-kinase (*PI3K*) activity (Wong et al., 2019), an enzyme that is involved in cellular growth, proliferation and differentiation, and which has been previously been associated with SCZ (Law et al., 2012).

Brain-derived Neurotrophic factor (*BDNF*) is essential for neurogenesis and extensively studied as a biomarker in psychiatry (Lin & Huang, 2020) There is extensive evidence of a difference in DNAm in *BDN*F, as well as EWAS studies showing enrichment for the neurogenesis pathway in SCZ (Di Carlo et al., 2019; Ursini et al., 2016), BD (Dell et al., 2014; Duffy et al., 2019), PTSD (Kim et al., 2017; Uddin et al., 2010), Depression (Hing, Sathyaputri, & Potash, 2018; Kang et al., 2015), Borderline PD (Arranz et al., 2021; Thomas et al., 2018), Anxiety Disorders (D'Addario et al., 2019), ED (Thaler et al., 2014), ASD (Ramaswami et al., 2020) thus making a well-replicated epigenetic transdiagnostic finding in psychiatry.

As a whole, transdiagnostic evidence suggests an involvement of DNAm changes in neurogenesis an neural plasticity.

### Immune system and inflammation

Abundant evidence supports the role of neuroinflammation and altered immune processes in the aetiopathogeneses of various mental conditions (Mazza, Lucchi, Rossetti, & Clerici, 2020; Pariante, 2017).

An EWAS by Montano et al. (Montano et al. 2016) found differences in DNAm in genes involved in T-cell development in the blood of SCZ patients (*ZC3H12D, TCF3*, and *IKZF4*); other EWAS have also reported an enrichment in the immune system pathway by differently methylated genes (*FR2B, PIK3R3, INPP5D, FCGR2C, IGHA1, FCAR; CD224, LAX1, TXK, PRF1, CD7, MPG,* and *MPOG*) (Aberg et al., 2014; Hannon et al., 2016; Liu et al., 2014).

In depression, a discordant monozygotic twin study based on peripheral blood, found 39 DMRs associated to a lifetime history of MDD, which were significantly enriched in biological pathways associated to cytokine secretion (Zhu et al., 2020). Another EWAS on post-mortem brain of people with late-MDD (Hüls et al., 2020), found altered DNAm in the *YOD1* locus, which is dysregulated in depression (Howren, Lamkin, & Suls, 2009) and its implicated in the regulation of inflammatory processes (Schimmack et al., 2017).

Two EWAS studies in PTSD found differences in DNAm across genes part of biological pathways involved with inflammation and immune response (Kuan et al., 2017; Uddin et al., 2010).

In ASD, various EWAS studies have pointed at dysregulation of pathways related to immune response (Ramaswami et al., 2020; Wong et al., 2019), and in a genome-wide microRNA (miRNA) expression profiling study (Wu, Parikshak, Belgard, & Geschwind, 2016).

Furthermore, an EWAS study from patients suffering from Panic Disorder found enrichment in genes involved in the regulation of lymphocytes (Shimada-Sugimoto et al., 2017).

We can conclude that there is transdiagnostic, rather than specific, a link between DNAm changes in the immune system and inflammation and neural plasticity, although evidence is more robust in SCZ.

### Oxidative stress

There is converging evidence pointing at a role of redox dysregulation as a possible mechanism involved in the aetiopathogenesis of both ASD (Bjørklund et al., 2020) and psychosis (Perkins, Jeffries, & Do, 2020). Oxidative stress has been shown to play a role in epigenetic modifications, enhancing inflammatory gene transcription (Rahman, Marwick, & Kirkham, 2004). Oxidation of 5mC to the 5-hydroxymethylcytosine (5hmC) is considered a key step in the reversibility of DNA methylation, which can have important therapeutic implications. Moreover, glutathione, the major antioxidant in the brain, is involved in the methionine cycle, and depletion of glutathione can be detrimental for the DNAm process (García-Giménez, Roma-Mateo, Perez-Machado, Peiro-Chova, & Pallardó, 2017).

Although evidence examining this pathway in the context of epigenetics is scarce, some EWAS have shown interesting results: one study examined prospectively the association of EWAS methylation changes with the transition to psychosis (Kebir et al., 2017), and found an enrichment of pathways related to oxidative stress regulation in those transitioning. Furthermore, an EWAS study in Borderline PD found differences in methylation in *GCT6*, which is important in glutathione metabolism (Arranz et al., 2021).

## The epigenetic signature of childhood adversity and cannabis use

The characteristic of the studies described in this section can be found in Online Supplementary Table S1 (SM), and in Table 2.

### Hypothalamus pituitary-adrenal axis pathway

While multiple studies have explored the link between epigenetic modification involved in the HPA-axis, and psychiatric disorders, recent evidence is beginning to indicate that some of these epigenetic modifications might follow exposure to CA. The latter is a robustly replicated risk factors for many psychiatric disorders (Online Supplementary Table S1 (SM) summarises the main findings on studies examining the link between DNAm and genes involved in the HPA-axis, and key findings are summarised in Fig. 2). As a whole, as illustrated in Fig. 2, at the level of *NR3C1* there is consistent evidence on a positive correlation between CA and DNAm in Borderline PD and MDD and some clinical outcomes (Farrell et al., 2018; Martin-Blanco et al., 2014; Perroud et al., 2011; Radtke et al., 2015), and a negative correlation with anxiety and PTSD (Labonte, Azoulay, Yerko, Turecki, & Brunet, 2014; Schechter et al., 2015; Wang et al., 2017; Yehuda et al., 2015). Lower DNAm in *FKBP5* is associated with CA in psychosis and PTSD (Klengel et al., 2013; Misiak et al., 2020); while in depression, 3 studies found no such link (Bustamante et al., 2018; Farrell et al., 2018; Klinger-König et al., 2019), as opposed to another study (Tozzi et al., 2018). As for NR3C1, findings on *FKBP5* DNAm are different across disorder, suggesting a divergent transdiagnostic mechanism involving in HPA related genes (see Fig. 2). The *SKA2* interacts with adversity scores in predicting lifetime suicide attempt (Kaminsky et al., 2015), and mediated the association between reduced cortical thickness and PTSD (Sadeh et al., 2016a) and suicide phenotypes (Sadeh et al., 2016b).

### Serotoninergic, dopaminergic and glutamatergic/GABAergic pathways

#### Childhood adversity

With regards to the serotoninergic pathway, while hypomethylation in *SLC6A* is associated with resilience to PTSD (Koenen et al., 2011), hypermethylation of *SLC6A* has been linked to exposure to CA and associated with the worst clinical presentation in MDD (Kang et al., 2013). Moreover, hypermethylation in the *5-HT3A-R* gene appeared to mediate the link between exposure to adversity and higher severity of disease parameters in a mixed sample of BD and Borderline PD (Perroud et al., 2016).

Moreover, hypomethylation of *MAOA*, a gene important for the degradation of serotonin and DA (Xu, Jiang, Gu, Wang, & Yuan, 2020), appears to partially mediate the known association between CA and depressive symptoms, alongside with other stress-related genes such as BDNF and NR3C1 and SLC64 (Peng et al., 2018). Moreover, *MAOA* DNAm was negatively correlated to life events in a sample of Panic Disorder (Domschke et al., 2012).

In relation to DA, one study in patients with bulimia spectrum disorders found no differences in *DRD2* DNAm when comparing those with exposure and non-exposure to trauma (Groleau et al., 2014).

At the level of the Glutamatergic pathway, one study found that exposure to CA was associated with decreased DNAm in *GAD* in a sample of Panic Disorder (Domschke et al., 2013). Lastly, a candidate gene study (Engdahl, Alavian-Ghavanini, Forsell, Lavebratt, & Rüegg, 2021) and an EWAS (Weder et al., 2014) linked CA to increased methylation levels in *GRIN2B/GRIND1* genes, suggesting evidence that this change may lead to the onset of depressive symptoms.

As a whole, DNAm changes in some of the genes that have been previously linked to major psychiatric conditions (Section ‘Evidence of epigenetic processes in major transdiagnostic pathways’, Table 2), such as *SLC6A*, *5HT3A-R*, *A* (*MAOA*), *BDNF*, *GAD* and the *GRIND* family, (related to the serotoninergic, and glutamatergic pathways respectively) are also associated to CA. This suggests that some of the DNAm changes attributed to these disorders may be partially related to the consequence of CA exposure, as illustrated in Fig. 1.

#### Cannabis use

CU and in particular heavy use (Marconi et al., 2016) has been consistently associated with increased risk for PD, but to a lesser degree for other psychiatric conditions (Sideli, Trotta, Spinazzola, La Cascia, & Di Forti, 2020b). In recent years, candidate genes studies from peripheral blood have reported changes in DNAm associated with heavy CU in genes involved in dopamine transmission, such as *DRD*2 (Gerra et al., 2018), *DAT1* (Grzywacz et al., 2020) and *COMT* (Van der Knaap et al., 2014) and in the *CB1* and *CB2* receptors genes part of the endocannabinoid system (Rotter et al., 2012; Tao et al., 2020). The latter playing an important role in brain development and synaptic transmission.

A recent study investigated the effect of heavy CU with and without tobacco on EWAS (Osborne et al., 2020). The analyses in the sample with both cannabis and tobacco use identified differentially methylated sites in 2 genes, *AHRR and F2RL*, previously reported to be affected by tobacco exposure. Within the sample of cannabis users without tobacco, while none of the differentially methylated loci reached EWAS significance, an exploratory analysis showed enrichment for genes involved in the signalling pathway, including glutamatergic transmission, brain function and mood disorders. Moreover, these exploratory analyses show two differentially methylated sites significantly associated with both only CU and cannabis with tobacco, which are within the *MARC2* gene. The latter previously linked to adverse effects to antipsychotics in schizophrenia (Åberg et al., 2010) and within the *CUX1* gene which is involved in neuronal development (Platzer et al., 2018).

Furthermore, recent whole blood and cell-specific Methylome-wide association studies (MWAS) from a sample of adolescents with CU disorder pointed at many methylation sites relevant to brain function and to neurodevelopment (Clark et al., 2021). These included CpGs located in the *CLMN* gene and the *SENP7* gene, expressed in the brain and playing a role in brain developmental and synaptic function and organisation (Juarez-Vicente, Luna-Pelaez, & Garcia-Dominguez, 2016; Marzinke & Clagett-Dame, 2012). Interestingly, the pathway analyses based on the cell type-specific significant DNAm changes associated with CU implicated pathways such as the Slit-Robo signalling (granulocytes) under the regulatory control of the endocannabinoid system during brain cortical development (Alpár et al., 2014), the *ErbB* signalling pathway (T-cell) and pathways involved in DNA repair (B-cell) (Clark et al., 2021).

### Inflammation, oxidative stress, synaptic plasticity and neurogenesis

#### Childhood adversity

A number of EWAS studies conducted in clinical samples have reported an association between exposure to CA and DNAm changes across genes involved in inflammation. For instance, a study (Prados et al., 2015) found a positive correlation between the *IL17RA* DNAm and CA in a Borderline PD and MDD sample. Other evidence suggests a negative correlation between DNAm in genes enriched for immune pathways (such as *TLR1* and *TLR3*) and CA in PTSD subjects (Uddin et al., 2010); while the *TNFRSF13C* gene was differently methylated between Borderline PD participants with and without CA (Arranz et al., 2021) (See Online Supplementary Table S1 (SM) – EWAS section).

Candidate genes studies have linked CA with DNAm changes in *BDNF* (Moser et al., 2015; Thaler et al., 2014; Weder et al., 2014), consistently with EWAS data reporting DNAm changes affecting genes involved in neurogenesis (Prados et al., 2015; Uddin et al., 2010). For instance, three EWAS studies in BD (Comes et al., 2019), Borderline PD (Arranz et al., 2021) and MDD (Lutz et al., 2017) have consistently shown changes in DNAm in genes from the *POU* family that are associated with CA (*POU6F2*, *POU5F1* and *POU3F1* respectively), which are genes involved in myelinisation and neurogenesis (Online Supplementary Table S1 (SM) – EWAS section).

A recent EWAS study found differences in DNAm of the *GGT6* gene that were associated with exposure to CA in a sample of Borderline PD patients; *GGT6* is key for glutathione homoeostasis (Arranz et al., 2021), it is also the main antioxidant and redox regulator that has previously been associated with SCZ aetiopathogenesis (Steullet et al., 2016). Further evidence is summarised in Online Supplementary Table S2 (SM).

As a whole, candidate gene and EWAS studies suggest a link between CA and genes involved in the inflammatory and neurogenesis pathways, with some preliminary evidence suggesting a link between CA and DNAm and oxidative stress genes (Fig. 1).

#### Cannabis use

The largest to date case–control study to examine the effect of lifetime CU on DNAm reported an epigenome-wide-significantly differentially methylated CpG site within *the CEMIP* gene (Markunas et al., 2020). The *CEMIP* gene, involved in hyaluronic catabolism, which has been shown to have an important role in inflammation, immune processes as well as associated with BD and SCZ previously (Petrey & de la Motte, 2014).

## Other environmental exposures that can act as confounders in psychiatric epigenetic

### Tobacco smoking

A number of publications have identified robust associations between tobacco smoking and DNAm (Elliott et al., 2014; Shenker et al., 2013; Tsaprouni et al., 2014; Zeilinger et al., 2013), with a number of genes (e.g. *AHRR*, *F2RL3*, *GFI1* and *MYO1G*) replicated across studies.

The confounding effect of smoking is clearly evidenced in an EWAS study on peripheral blood of SCZ patients (Hannon et al., 2016). A similar study examining the impact of CA on the epigenome in a general population found that tobacco consumption was an important confounding when examining the signature of CA (Marzi et al., 2018). Whether some of these epigenetic changes associated with tobacco exposure could also mediate the already reported link between tobacco use and increased risk of psychosis, it is an important question yet to be determined (Gurillo et al., 2015), and tobacco smoking should be accounted for in the future epigenetic studies in psychiatry.

### Alcohol use and abuse

There is some initial evidence to suggest that alcohol use is associated with DNAm changes (Liu et al., 2016; Wang, Xu, Zhao, Gelernter, & Zhang, 2016; Weng, Wu, Lee, Hsu, & Cheng, 2015). Enrichment analyses examined DNAm in alcohol users have revealed enrichment in pathways related to neural degeneration (Weng et al., 2015), and in genes important for neurogenesis (*NPDC1*), inflammation (*HERC5*) and in GABA receptors (a receptor delta and B receptor subunit 1); all of which are pathways previously associated with different mental disorders, as shown in Fig. 1. However, studies rarely account for such covariates, which is currently a limitation of current literature.

### Psychiatric medication

The extent of the data reporting the DNAm changes associated with psychiatric medication would require a separate review. Indeed, there is consistent evidence that pharmacological agents can trigger DNAm in similar or opposite directions than those attributed to the disease. For example, Lithium, Carbamazepine and Quetiapine, often prescribed for the treatment of BD, are associated with decrease methylation of *SLC6A4* (Asai et al., 2013; Sugawara et al., 2015), in contrast with the hypermethylation reported in BD in that gene (Table 2). Similarly, studies who have investigated the effect of antipsychotic medication, have shown, on the one hand, that Haloperidol affects DNAm in leucocytes of SCZ patients (Melas et al., 2012), while on the other hand, a recent EWAS study showed that Clozapine exposure leads to DNAm differences in patients with treatment-resistant SCZ (Hannon et al., 2021) as compared to controls. Thus, it is key to consider the possibility that some of the changes in DNA pathways may be led by agents rather than the disease itself, highlighting the need to account for medication in future studies and to consider epigenetics as a potential mediating mechanism of action of the beneficial effects of medication in the brain.

## Summary and outstanding questions

As illustrated in Fig. 1, many of the epigenetic dysregulations we report are transdiagnostic, such as those affecting the serotoninergic, inflammatory and neurogenesis pathways, while others such as the Glutamatergic/GABAergic pathway are shared between a couple of disorders (e.g. SCZ and ASD), or disorder specific such as the dopaminergic pathway in PDs. These are pathways that have been classically implicated in the aetiopathogenesis of psychiatric phenotypes; additional emerging pathways such as oxidative stress remain to be further explored.

Moreover, CA, is transdiagnostically associated to psychiatry morbidity, and seems to play a role in the DNAm dysregulation of many of these pathways. Furthermore, the preliminary DNAm changes so far reported associated with CU affect pathways previously link to psychosis, suggesting potential mediating venues to be tested in clinical populations (Fig. 1).

In addition, CA is associated with DNAm changes both in the general population (Kandaswamy et al., 2020) as well as in clinical samples with a psychiatric diagnosis (Online Supplementary Table S1 (SM)). This might suggest that the DNAm changes associated to CA exposure predate disease onset and could represent a marker of acquired psychiatric liability. However, evidence formally testing mediating pathways EWAS level between CA and the main clinical conditions is non-existent in humans. Candidate gene studies tend to find the inconsistent direction of the association between CA across disorders, or findings are inconsistent within disorders as shown in Fig. 2 and Table 2 and Online Supplementary Table S1 (SM). One explanation could be that there are other causative partners that are being missed in the equation, that may explain such inconsistency, such as the role of genotype, gene expression or a more thorough assessment of specific adversities in the context of protective factors and its link with more carefully selected clinical phenotypes.

The existing findings from epigenetics research need to be appraised in the context of well-known technical limitations epigenetics, such as the blood-brain inconsistencies, tissue-type specificity (Bakulski, Halladay, Hu, Mill, & Fallin, 2016; Nikolova & Hariri, 2015) and the candidate gene *v.* EWAS issue (see Palma-Gudiel, Córdova-Palomera, Leza, & Fañanás, 2015). Moreover, evidence suggests that variation in DNAm depends not only on the environment, but also on genetic factors (Bell & Spector, 2012). Although some studies presented in this review have found evidence that some genotypic variation in some risk alleles can influence DNAm (Klengel et al., 2013; Melas et al., 2013; Perroud et al., 2016), EWAS addressing the joint effect of genotype and environment are still in its infancy (Min et al., 2021). Addressing this issue will prove methodologically challenging, but methods quantifying the genetic influences on DNAm, such as the methylation quantitative trait loci (mQTL) should be used in relation to the presence of environmental insults. Moreover, studies included in this work are often small (Online Supplementary Table S1 (SM)) and thus underpowered, except some exceptions (Hannon et al., 2021), which presents the need to create collaborative efforts allowing meta-analysis of comparable epigenetic data. Furthermore, evidence of the environmental exposure impact through epigenetic modification in psychiatric diseases or phenotypes is still limited, with studies focusing mainly on exposure to CA and only preliminary results of the effect of cannabis. Given the replicated but differential impact of multiple environmental risk factors in major psychiatric disorders (Rodriguez et al., 2021), future studies exploring epigenetic variation as a mediator between genetic vulnerability and various environmental factors (not only CA) should be addressed, using novel methods specifically developed for mediation using EWAS data (Liu et al., 2021). Another important factor is the phenotypic characterisation for environmental exposure. For instance, most of the studies in this work have used broad measures of adversity, using a composite cumulative score, rather than differentiating between neglect of abuse. The same can be said for the measures of CU which little reflects the level of exposure none to affect psychiatric liability. Moreover, the outcomes are often considered as SCZ, or MDD or even major psychoses (combining SCZ & BD), which are extremely heterogeneous entities, involving microphenotypes (Maj et al., 2021), and which accordingly may have very different biological underpinnings. Evidence is showing that there are some levels of specificity between adversity subtypes and symptoms domains, for example, abuse is more related to positive symptoms while neglect is not (Alameda et al., 2021) and that CU is associated with paranoia (Freeman et al., 2005). Thus, using a composite measure of CA and broadly defined conditions when trying to understand specific mediating epigenetic pathways may consider such specific links between environment and psychopathology first. Accordingly, this work suggests that some biological pathways are operating transdiagnostically, and therefore a phenotypic characterisation based on clinical dimensions may be more biologically informative than diagnostic categorisations. Furthermore, the timing of environmental exposure should be addressed, given evidence that a disruption in epigenetic programming occurs across different time windows throughout the life span (Massicotte, Whitelaw, & Angers, 2011). In this line, the lack of information on the timing of trauma and of CU initiation could explain some of the inconsistencies mentioned in our review (Fig. 2). For example, we reviewed studies showing increased methylation of the serotonin transporter in depressed individuals exposed to trauma (usually when adversity occurs before adulthood), which contrasts with the lower methylation in the same gene in PTSD, when exposure tends to be later in life.

## Conclusions

Future Research should include the influence of gender and how it can modulate the links between DNAm and mental disorders, or how it can affect the influence of CA on DNAm. More effort should go towards designing studies that integrate genetic data with the often-neglected effect of environmental exposures (e.g. recreational drugs and psychotropic medication). Specifically, collaborative efforts between geneticists, epigeneticists and epidemiologists will lead to increased understanding of how the DNAm changes mapping to specific pathways, might mediate the biological link between environmental exposures and increased liability to specific or transdiagnostic psychiatric morbidity.

## Supplementary material

For supplementary material accompanying this paper visit https://doi.org/10.1017/S0033291721005559. click here to view supplementary material
