# Supplementary material for: Can epigenetics shine a light on the biological pathways underlying major mental disorders?
Source: Psychol Med. 2022 Feb 23;52(9):1645–65. doi: 10.1017/S0033291721005559 (PMC9280283; doi:10.1017/S0033291721005559)
Supplement: Supplementary file 1 [file S0033291721005559sup001.docx]

**SUPPLEMENTARY MATERIAL**

**Supplementary Table 1.** Studies exploring the interplay between childhood adversity, DNA methylation and clinical features

| **Author year** | **Sample size**  **Gender (M/F)**  **Ancestry or population** | **Design** | **Measure of adversity** | **Objective** | **Tissue** | **Biological Pathway**  **(gene)** | **Diagnosis/**  **psychopathology measure** | **Analyses/**  **Confounders** | **Main**  **Findings** |
| --- | --- | --- | --- | --- | --- | --- | --- | --- | --- |
| **Candidate genes studies** | | | | | | | | | |
| **Hypothalamic Pituitary Adrenal Axis studies** | | | | | | | | | |
| **(Misiak et al., 2020)** | 85 inpatients with SCZ  56 HC  SCZ: 45M/40F  HC: 22M/34F  Polish sample | Cross-sectional | CECA | (i)Examine *FKBP5* methylation in patients with FEP, acutely relapsed SCZ (SCZ-AR) patients,  and HC. (ii) Investigated the association between clinical manifestation, cognitive performance and a history of CA in this group of patients. | Blood (leukocytes)BC + PCR +PYR  Pyrosequencing | 4 CPGs *FKBP5* | Diagnoses: (OPCRIT)  Psychosis: (PANSS)    Depression: (MADRS)  Mania: (YMRS)    Functioning: (GAF)  Cognitive performance  (RBANS) | Analyses of co-variance  Age, sex,  body mass index (BMI), cigarette smoking status, cortisol levels and CPZ (chlorpromazine) equivalent | Lower methylation in *FKBP5* CpG4 (and not CpG1, 2 and 3) related to parental antipathy and sexual abuse.  (ii) Lower DNAm of FKBP5 associated to better cognitive performance in patients, with an opposite pattern in HC. |
| **(Bustamante et al., 2018)** | N= 112  (56 with MDD; 56 without MDD)  50M/62F  European American/African American /other | Cross-sectional  Case-control (nested) | Adaptation of CTS and the CTQ  Composite (CA) | (i) Whether *FKBP5* DNAm mediates CA-Depression | Blood  BC + PCR +  Pyrosequencing | FKBP5 | Depression symptoms severity  (PHQ-9) | Mediation (B & K) with regression-based approach  Age, sex, race, PBMC count,  medication | (i) Null mediation  (Path CA-DNAm was not significant) |
| **(Klinger-König et al., 2019)** | N=3965 general public  48% Male (2078M/11887F)  Caucasian sample | Cross-sectional | CTQ | (I) to test main effects of CA,  MDD, and the SNP rs1360780 on  DNAm of five CpG sites of intron 7 of the  FKBP5 gene, and the mean DNAm of all CpGs  (ii) to test the two-way interactions (CA x MDD, CA x SNP,  MDD x SNP) and the three-way-interactions (CA x depression x SNP) on FKBP5 DNAm were tested | Blood  MALDI-TOF mass spectrometry using  EpiTYPER by MassARRAY | Five CpG sites in intron 7 of the FKBP5 gene | Lifetime MDD  (M-CIDI)  Severity of depressive symptoms  (BDI-II)  Lifetime MDD: PHQ-9 | 2- and 3-way interactions effects  Smoking, waist circumference, white and red blood cell counts,  platelet counts and mean platelet volume, fibrinogen, glycated  haemoglobin, cystatin C, triglycerides and time of blood sampling  on the methylation levels of the five CpG sites were tested | No main or interaction effects found. |
| **(Tozzi et al., 2017)** | Sample 1  31MDD/29HC  HC:17F/12M  MDD:21F/10M  Sample 2  25MDD/21HC  HC:13F/8M  MDD:15F/10M  Irish sample | Cross-sectional | CTQ | (i) whether CA is associated with FKBP5 DNAm  in MDD and controls  (ii) whether FKBP5 DNAm is associated  with structure and function of emotional processing regions. | Blood  BC + PCR +  Pyrosequencing | CG-6 and CG-7 in the  intron 7 region of FKBP5 | The Hamilton Depression Scale (HAM-D) 21-item version  (Hamilton, 1986), Beck’s Depression Inventory (BDI) | General linear model (GLM)  Correlations  Age, sex, site, and medication | (i) negative correlation  CTQ/FKBP5  (ii) lower FKBP5 DNAm associated to reduced grey matter in frontal orbital gyrus bilaterally |
| **(Klengel et al., 2013)** | N= 76  Trauma subjects: 30  Non Trauma: 46  22M/8F  36M/10F  Predominantly African American | Cross-sectional | CTQ and TEI | *To test whether FKBP5* (genotype) × CA interactions are mediated by epigenetic modifications | Blood  BC + PCR + DNAm Validation Using Sequenom’s EpiTYPER MassARRAY | FKBP5 | Current/lifetime PTSD diagnosis _ symptoms  (mPSS and CAPS) | Logistic regression  Age and gender, current depressive symptom/CA  Bonferroni correction | Demethylation of the FK506 mediate the combined effect of CA and the *FKBP5* risk allele on PTSD |
| **(Bustamante et al., 2016)** | N=147  (73 with MDD and 74 without MDD)  56M/91F  European American/African American /other | Cross-sectional  Case-control (nested) | Adaptation of CTS and the CTQ  Composite (CA) | (I) CA & PHQ-9 interaction on NR3C1  (ii) whether NR3C1 differences are associated to gene expression (not detailed) | Blood  BC + PCR +  Pyrosequencing | NR3C1 | Depression symptoms severity  (PHQ-9) | Linear regression  Age, sex, race, PBMC count,  medication | (i)No significant CA & PHQ-9) interactions were observed.  Trauma alone was associated with significantly lower NR3C1 GE. |
| **(Farrell et al., 2018)** | N=33 MDD/34 Controls  21M/45F | Cross-sectional | CTQ | Associations between CA ad (NR3C1) exon 1F and  the FKBP5 gene intron 7 | Blood  BC + PCR +  Pyrosequencing | NR3C1 exon 1F and the FKBP5 gene intron 7 | MDD:  (M.I.N.I)  + scored >  17 on the HAM-D-21 | Correlation analyses  (no adjustment in CTQ/methylation) | DNAm levels at specific CG sites within the NR3C1 exon 1F were related to childhood emotional abuse  severity. (positive association)  FKBP5 no association |
| **(Tyrka et al., 2016)** | N= HC 340 adults  (147 CA+;193 CA-)    CA+:59M/88F  CA-: 68M/125F  78% white | Cross-sectional  Population based | CTQ + interview  Composite | (i)impact of psychopathology/CT on NR3C1 1promote region  (comparing means between 4 groups) | Blood  Leucocytes  BC + PCR +  Pyrosequencing | NR3C1 1promote region | Anxiety, depressive and substance use disorders  SCID | General linear models  Age, sex and oral contraceptive use (excluded patients on psychotropic medication) | (i)Number of adversities was negatively associated with NR3C1 methylation in participants with no  lifetime disorder but not in those with a lifetime disorder |
| **(Perroud et al., 2011)** | N: 230 patients (101 Borderline PD; 114 MDD subjects without PTSD; 15 MDD with comorbid PTSD)  Borderline PD: 6M/95F  MDD without PTSD: 35M/64F  MDD with PTSD: 4M/11F  Suisse/French sample  (NA race) | Cross-sectional  Clinical sample | CTQ  composite | (i)Impact of CA on NR3C1 promote region | Blood white cells  BC + PCR +Pyrosequencing | exon 1F NR3C+ promoter | Depression and PTSD: French-DIGS  Borderline PD:  Screening Interview for Axis II Disorders Borderline PD part | Linear regression  Gender, primary Axis  I diagnosis, alcohol/substance use; PTSD, history of suicide attempt, severity of  depression and of Borderline PD and current medication | Childhood sexual abuse, its severity and the number of type of maltreatments positively correlated with NR3C1 methylation  In Borderline PD, repetition of abuses and sexual abuse with penetration correlated with a higher methylation percentage |
| **(Wang et al., 2017)** | N=149  64 GAD and 85 HC  HC: 28/57  GAD: 19/45  HC (Han/other)  82/3  GAD  63/1  Chinese population | Cross-sectional | CTQ | (i) link NR3C1 1F promoter methy-clinical parameters (including CA) | Blood  PMBCs  BC + PCR | NR3C1 1F promoter | GAD diagnosis DSM-IV axis I + (HAM-A) | Correlation analyses  Multiple comparisons  None | Overall, NR3C1 1F Meth  increased and the mRNA level of GR decreased in the  PBMCs in patients with current GAD compared with healthy controls  Negative correlation of overall methylation of the NR3C1 1F promoter with its mRNA level in the PBMCs  CA tended to induce lower  methylation in the NR3C1 1F promoter |
|  |  |  |  |  |  |  |  |  |  |
| **(Steiger, Labonté, Groleau, Turecki, & Israel, 2013)** | N= 96    64 BN; 32 HC  Female sample  Canadian sample | Cross-sectional  Case control (Nested) | CTI | (i) compare NR3C1 prom meth between BN and HC  (ii) in BN impact of CA/suicidality/Borderline PD on NR3c1 prom meth. | Blood Lymphocytes  BC + PCR | NR3C1 | -EDE for BN, -SCID-I and  DIS4 for comorbid DSM-IV axis I diagnosis.  CAPTSDI for PTSD  SCID-II for comorbid axis II diagnosis  axis II diagnosis | ANOVA/ANCOVA  Binge frequency, vomit frequency, BMI, medication | (i) more meth in women with BN and or Borderline PD/suicidality vs HC  (ii) none effect of CA |
| **(Rachel Yehuda et al., 2015)** | N: 122 veterans  61 PTSD  61 Non PTSD (resilients)  Male sample  47% Hispanic  26.2% Non-Hispanic white  26.3 Non hispanic back | Case-control  (Nested) | ETI | NR3C1-1F promoter meth in PTSD versus Non-PTSD veterans (resilients) | Blood  PBMCs  BC + PCR | NR3C1-1F promoter | PTSD diagnosis (CAPS) | Bivariate and partial correlations  PBMC ratio as covariate | Lower NR3C1-1F promoter methylation in PTSD veterans |
| **(Daniel S Schechter et al., 2015)** | N= 45  IPV-PTSD: 28  Controls: 17  Female  Swiss sample | Cross-sectional | (BPSAQ)  (TLEQ) | Link between CA and NR3C1 1F meth and its inverse correlation with PTSD | Saliva  BC + PCR + Pyrosequencing | NR3C1 1F | Current/lifetime PTSD diagnosis symptoms  (PCL-S and CAPS) | Correlations  Bonferroni corrections | Maternal PTSD severity and parenting stress were negatively correlated with the mean % NR3C1 DNAm, which was associated with mPFC activity in the mother and poorer cooperativeness in the child |
| **(Labonte, Azoulay, Yerko, Turecki, & Brunet, 2014)** | N=46  PTSD= 30  Controls (non trauma) =16  PTSD 15M/15F  Controls (8M/8F)  Canadian sample | Cross-sectional | Index traumatic events | To test whether HPA axis alterations in PTSD are associated with variations in  cortisol levels due to changes in NR3C1 expression | Blood Lymphocytes  BC + MALDI-TOF mass spectrometry using  EpiTYPER by MassARRAY | NR3C1 | PTSD: CAPS | Mixed model analysis  Correlations  Least significant difference post hoc  tests  Age, sex. | Significant overall and site-specific hypomethylation  in NR3C1 promoter (1B and 1C) in the PTSD group  compared to controls |
| **(Radtke et al., 2015)** | N=46 from general population  28F/18M  German sample | Cross-sectional | MACE German version | (i) whether the simultaneous  occurrence of ELS and increased NR3C1 DNAm is  accompanied by an increased vulnerability to the development of  psychopathology. | Blood Lymphocytes  GWAS  The Human Methylation 450 K array + BC | All probes spanning the NR3C1 gene, which are  included on the Human Methylation 450 K array  (41 probes) | BSL- 23  HSCL  MINI  KIDSCREEN-53 | Multiple-regression analyses  Correlations  None | Additive effect of childhood maltreatment and NR3C1 DNAm predicting borderline personality disorder (Borderoline PD)-  associated symptoms |
| **(R. Yehuda et al., 2016)** | N= 122 combat veterans with similar level of CA (61 PTSD; 61 Non-PTSD)(same amount of adversity)  Male sample  PTSD  Hispanic 47.5%  Non-Hispanic White 26.2%  Non-Hispanic Black 26.2%  Non-PTSD  Hispanic 32.8%  Non-Hispanic White 37.3%  Non-Hispanic Black 26.2% | Cross-sectional | ETI | (i) DNAm NR3C1-1F promoter between PTSD and non-PTSD  (ii)Testing whether NR3C1-1F promoter DNAm would be inversely associated with NR3C1-1F  expression and PTSD clinical outcomes | Blood  BC + Pyrosequencing | NR3C1-1F | PTSD with CAPS) | A multivariate ANOVA  Bivariate and partial correlations  PBMC ratio | (i) lower NR3C1-1F promoter DNAm in PTSD vs Non-PTSD veterans    (ii) NR3C1-1F promoter DNAm  correlated  PTSD-related symptoms (distress,  dissociation, and poorer sleep) |
| **(Martin-Blanco et al., 2014)** | N = 281 Borderline PD  42/239  Caucasian of European descent | Cross-sectional | CTQ-SF | Association between NR3C1 methylation status, the history of CT, and current clinical severity in subjects with Borderline PD | Blood  Leukocytes  BC + PCR + Pyrosequencing | NR3C1 | Borderline PD (SCID-II, DIB-R) | Linear regression models | NR3C1 methylation in subjects with Borderline PD may be associated not only with CT but also with clinical severity |
| **(Kaminsky et al., 2015)** | 421 (general population discovery sample)  299F/421M  93.1% African American ancestry | Cross-sectional | CTQ  and TEI | (i)methylation SKA2 in relation to CTQ  (II)CTQ vs SKA2meth on suicide attempt/PTSD | Blood (replication saliva N=61)  Illumina (San Diego, CA,  USA) HumanMethylation450 BeadChip  PCR + Pyrosequencing | SKA2 methylation | Anxiety symptoms were assessed as a continuous score from the (HAM-A). | Linear regression  analysis  Age, sex and race | (i)SKA2 methylation interacted with CTQ scores to predict lifetime suicide attempt in saliva and blood  (ii)SKA2-meth predicted PTSD caseness when CTQ was considered |
| **(Sadeh, Spielberg, et al., 2016)** | 200 trauma-exposed veterans  182/18  White non-Hispanic | Cross-sectional | CAPS | Associations between SKA2 methylation, cortical thickness and psychiatric phenotypes linked to suicide | Blood  BC + PCR + HumanMethylation450 K microarrays | SKA2 | Current depression symptom severity was assessed was assessed via the14-item total depression subscale of the self-report depression anxiety stress scale  MDD (SCID-I) | Hierarchical linear regression models + Mediation analyses  Genotype | PTSD symptom severity was positively correlated with SKA2 DNAm. Mediation analyses showed a significant indirect effect of PTSD on cortical thickness via SKA2 methylation status. |
| **(Sadeh, Wolf, et al., 2016)** | 466 trauma-exposed veterans and intimate partners  302/164  With non-Hispanic | Cross-sectional | CAPS | Associations between SKA2 DNAm, broad dimensions of psychiatric symptoms, and suicide phenotypes in adults with high levels of trauma exposure | Blood  BC + PCR | SKA2 | Internalizing and Externalizing Psychopathology (SCID-IV)  Suicide Phenotypes (Major depressive episode module of SCID-I) | Multiple regression analysis  Structural equation modelling  Hierarchical regression analyses  Age, sex, and the first three ancestry principal components | SKA2 methylation  may index a general  propensity to experience stress-related psychopathology, including internalizing  disorders and suicidal thoughts and behaviors. |
| **Studies on the oxytocin pathway** | | | | | | | | | |
| **(Simons, Lei, Beach, Cutrona, & Philibert, 2017)** | 100 (primary caregivers)  Female sample  100% African American ancestry | Longitudinal | Adult adversity (the four-item Unmet Material Needs Scale and the revised version of the community deviance scale developed for the Project on Human Development in Chicago Neighborhoods) | (i) mediation by methylation of OXTR between adversity and negative schemas and depression | Blood  BC + Illumina (San Diego, CA) HumanMethylation450 Beadchip | OXTR methylation | Mini-Mood and Anxiety Symptoms Questionnaire | SEM  Childhood  trauma, age, romantic relationship status, individual differences in cell types, and average level of genome-wide methylation | The effect of adult  adversity on distrust and pessimism is largely mediated by  methylation of OXTR. The effect of OXTR  methylation on depression is fully mediated by its association  with distrust and pessimism. |
| **(Smearman et al., 2016)** | 393 (general population)  70.7% Female  100% African American ancestry | Cross-sectional | CTQ  Traumatic Events Inventory | (i) assessing the role of OXTR methylation in the link between abuse and psychopathology | Blood  Omni-Quad 1M or the Omni Express BeadChip  (Illumina, San Diego, CA) | OXTR methylation | 21-item Beck Depression Inventory | Regressions or interactions  Age, sex, cellular heterogeneity, and  positional effects associate  with methylation of one or more OXTR CpG sites | Child abuse associated with higher methylation of two CpG sites yet did not  survive correction or serve as a mediator of psychopathology |
|  | | | | | | | | | |
| **Studies on the serotoninergic pathway** | | | | | | | | | |
| **(Domschke et al., 2012)** | N 65 PD (44F/21M)  65 HC (44F/21M)  100% Caucasian origin | Cross-sectional | Life events (comprehensive scale  derived from other life event scales) | (i) investigate DNAm changes associated to PD and its link with life events | Whole blood  BC + PCR | MAOA | Diagnosis of PD  on the basis  of medical records and structured clinical interviews  according to the criteria of DSM-IV | Correlations or regressions  Gender  stratification  Bonferroni’s correction  Age, smoking status and medication | In PD Negative life-events associated to decrease DNAm in MAOA at one CpG site; while positive life events associated with increased MAOA DNAm at another CpG. No such effect was found in controls |
| **(Kang et al., 2013)** | N=108 MDD  75% female  South Korean  population | Prospective  (12 weeks) | Questionnaire before age 16 including abuse and another adversities such as financial problems | (i) Link between  SLC6A4 DNAm and CA and clinical outcomes  At baseline and FU | Blood  BC + PCR | SLC6A4 | Depression  (HAMD), anxiety (HAMA), functioning (SOFAS), | Correlations  None | Higher SLC6A4 associated with CA and worst clinical presentation (SOFAS) at baseline only not at FU |
| **(Peng et al., 2018)** | 119 monozigotic twin pairs  29.5% female  USA sample | Cross sectional | ETISR-SF | (i) test mediation between CA and depression via stress related genes | Blood  Bisulfite pyrosequencing  Or  450K BeadChip. | BDNF,  NR3C1, SLC6A4, MAOA,  MAOB | Depression  (BDI-II) | Mediation analyses via regression models  Twin age, family income, cigarette smoking (pack-year), alcohol consumption, physical activity, BMI, and history of PTSD | BDNF and NR3C1 mediated ~20% of the association between CA and depressive symptoms |
| **(Melas et al., 2013)** | N:  Depression: 82  HC: 92  100% female  Swedish population | Cross-sectional  Case control (nested) | PART  Specific adversities (Familial constraints/problems;  Parental death/divorce) | (i) Link between Depression and MAOA methylation  (ii) Depression + CA types on NR3C1 1-F region methylation  (iii) mediation by  MAOA-L between parental death and NR3C1 1-F region methylation | Saliva Leucocytes  BC + PCR | MAOA  NR3C1 1-F region | Depression (MDI) | Linear regression  Age, diagnosis, parental death, financial problems, familial constraints, parental divorce, genotype, smoking | (i)Early parental death associated with increased DNAm at NR3C1;  (ii)MAOA-genotypic variations may affect the extent of NR3C1 methylation.  (iii)No association between EPD and depression |
| **(Perroud et al., 2016)** | N= 349  (Borderline PD: 116; ADHD: 111;  BD: 122)  Borderline PD: 10M/106F  ADHD: 78M/33F  BD: 57M/65F  European ancestry | Cross-sectional | CTQ | (i)Link between CA/5HT3AR DNAm and severity of the disease  (ii)Methylation mediates linkt between CA and psychopathology | Blood  BC + PCR | 5HT3AR | Borderline PD/ADHD (DIGS);  BD  (SCID)  Clinical index severity (admissions, suicide attempts, comorbid OH use etc..) | (i)Liner regression models  (ii)Mediation analysis  age, gender, and category of  diagnosis (i)  Bootstrapping (ii) | CA was associated with clinical severity and this was mediated by meth on two CPGs at 5-HT3A R |
| **(Daniel S. Schechter et al., 2017)** | N= 35  IPV-PTSD:18  Non-PTSD mothers (n=17)  Female  Swiss sample | Cross-sectional | (BPSAQ)  (TLEQ) | To explore link between HTR3A DNAm and maternal life stress, IPV and PTSD | Saliva  BC + PCR + Pyrosequencing | HTR3A | Current/lifetime PTSD diagnosis symptoms  (PCL-S and CAPS) | Correlations and multiple regressions  Bonferroni corrections | Methylation status of several CpG sites in the HTR3A gene was associated with maternal IPV and IPV-PTSD severity |
| **(Koenen et al., 2011)** | N=100  PTSD: 23  Non PTSD with 1 trauma (resilients): 77  Non PTSD: 32M/45M  PTSD:  8M/15F  79% African-American | Nested case-control study | Questionnaire on any traumatic events from a list of 19 (interpersonal and non- interpersonal) | To test whether SLC6A4 genotype and methylation  levels were associated with PTSD and modified the  effect of the number of traumatic events on risk for  PTSD | Blood (PBMC)  BC + PCR + Pyrosequencing | SLC6A4 | PTSD diagnosis  (PTSD checklist, delineated in three different ways) | Main and interaction effects in negative binomal logistic regression  age, PBMC count, number of  traumatic events, | Neither SLC6A4 genotype nor SLC6A4 meth were associated with PTSD  SLC6A4 meth  modified the effect of the number of CA on PTSD after controlling for  SLC6A4 genotype.  Persons with more CA but only at low SLC6A4 meth levels were at increased risk for  PTSD. |
| **Studies on Neurogenesis** | | | | | | | | | |
| **(Thaler et al., 2014)** | N=66  64 BN; 32 HC  Female sample  Canadian sample | Cross-sectional | CTI | Link BDNF DNAm- BN; Borderline PD CA | Blood Lymphocytes  BC + PCR + MALDI-TOF mass spectrometry using  EpiTYPER | BDNF | -EDE for BN, -SCID-I and  DIS4 for comorbid DSM-IV axis I diagnosis.  CAPTSDI for PTSD  SCID-II for comorbid axis II diagnosis | Multilevel modelling (MLM),  None | Women with BN showed  Increases BNDF DNAm  groups composed of individuals with CA or Borderline PD had particularly high levels of DNAm at selected CpG sites |
| **(Moser et al., 2015)** | N=46  20 control, 8 subthreshold IPV-PTSD  18 IPV-PTSD+  Female sample  Swiss Sample | Cross-sectional | BPSAQ | (i) explore the link between BDNF promoter DNAm and patterns of neural activity that are associated with maternal response to stressful  versus non-stressful child stimuli.  (ii) test  correlations between levels of IPV and BDNF meth | Saliva  BC + PCR + Pyrosequencing | BDNF | Lifetime PTSD: CAPS | Correlations  Monte Carlo simulation  None | (i) Severity of maternal anxiety was significantly correlated with overall DNAm of 4 CpG sites in exon IV of the BDNF promoter region  (ii) DNAm of CpG3 was associated with maternal exposure to domestic violence during childhood |
| **Studies on other pathways** | | | | | | | | | |
| **(Domschke et al., 2013)** | N=66 Panic disorder  65 HC  67% female  German population | Cross sectional twin study | Negative life events (not detailed) | (i) Linkin between DAD1/GAD2 and panic disorder  (ii) Linkin between DAD1/GAD2 and CA | Blood  BC + PCR | GAD1  GAD2 | Panic disorder  (SKID-I) | Correlation or regressions  Matched sample | (i) Patients with panic disorder had lower average GAD1  (ii) life events was associated with decrease DNAm GAD1. |
| **(Wolf et al., 2017)** | N= 339 PTSD  Male veterans  White non-Hispanic | Prospective 6.5 years after DNA collection and PTSD assessment | TLEQ | (i) link CA/PTSD-accelerated/decelerated epigenetic age  (ii) link DNAm age and mortality rates at FU | Blood  BC + PCR | Accelerated DNAm age | PTSD diagnosis (CAPS) | (i)Multiple regressions  (ii)Cox regression  Ancestry (top two PC), sex white blood cell count proportion | (i)No effect. Only Hypeearousal associated with DNAm age  (ii)DNAm age associated with 13% greater mortality at FU |
| **(Boks et al., 2015)** | N= 96 soldiers  32 low CA  67 high CA  Male soldiers  Dutch sample | Prospective study (pre-post deployment) | CTQ | (i) Prospectively investigated link DNAm accelerated age and trauma and  (ii) PTSD related symptoms at follow-up | Blood  BC + PCR  Illumina 450K DNA methylation  arrays | DNAm accelerated age | PTSD diagnosis (SRIP) | Linear regression analyses  Cell composition, alcohol, CA, excluding smokers and medicated subjects | (i)  CA significantly accelerated epigenetic ageing  (ii) Development of PTSD symptoms was inversely associated with epigenetic ageing |
| **(Sipahi et al., 2014)** | N= 60  30 PTSD  30 Non PTSD (resilients)  PTSD 7M/23F  Resilients 7M/23F  46 (77%)African-American  12(20%) Caucasian  2(1%) Hispanic | Prospective (approx. 1 year pre-post trauma) | Check list of interpersonal and non-interpersonal traumatic experiences | (i) explore pre trauma DNAm differences and whether changes during follow-up predict PTSD | Blood  BC + PCR + Pyrosequencing | DNA methyltransferase 1 (DNMT1), DNMT3A, DNMT3B and  DNMT3L | PTSD diagnosis was assessed via structured interview administered via telephone according to Breslau et al (1998) | Linear regression models  Bonferroni’s correction  Age, gender and pre-trauma symptom  severity | (i) DNMT1 DNAm increased following trauma in PTSD only  DNMT3A  and DNMT3B DNAm increased following trauma in PTSD/resilients  In PTSD only, pre-trauma DNAm was lower at a DNMT3B CpG  site  lower pre-trauma DNMT3B DNAm at this  site was predictive of worsening of PTSS post-trauma |
| **(Kimmel et al., 2016)** | N: 51 (66% MDD and 33% bipolar (I, II or NOS))  Female sample  John Hopkins cohort: 30% Caucasian/70% African American | Cross-sectional | Sexual abuse (clinical interview) | (i)To investigate if SA is associated with OXTR DNAm;  (ii) whether this effect is moderated by PPD status. | Blood  BC + PCR + Pyrosequencing | OXTR | MDD: Based on a psychiatric interview according to DSM-IV criteria | Correlation analyses  Linear regression  Bonferroni correction  None | SA status and PPD significantly interacted to modulate OXTR DNAm.  Post hoc analysis suggested that SA had independent effects on OXTR-Meth non-PPD women |
| **(Engdahl, Alavian-Ghavanini, Forsell, Lavebratt, & Rüegg, 2021)** | N=186 GP  121F/65M  Swedish sample | Cross-sectional | General questionnaire | (i) testing whether CA affects *GRIN2B* methylation in humans.  (ii) investigate if *GRIN2B* DNAm is associated with MDD | Saliva  BC + Pyrosequencing | GRIN2B | (MDI) Sheehan Patient-Related (Panic) Anxiety Scale | Linear regression models  Age, sex and CA | CA associated with increased GRIN2B DNAm  GRIN2B DNAm not associated with MDD status |
| **(Zou, Huang, Wang, Min, & Zhou, 2020)** | 113 patients with PD and 130 HC  PD  43M/70F  HC: 51M/79F  Chinese sample | Cross-sectional | CTQ | (i) explore whether inflammatory related genes DNAm may be associated with PD and severity of PD  symptoms,  (ii) explores the association of DNAm of these genes with  CA. | Blood  BC + PCR | 13 CpG region from the promoter regions of the inflammatory-  related genes, *CCL3_, CRP_,*  *CSF2_, CXCL8_, IFNG_1, IFNG_2, IL12B_, IL1A_, IL-4_, IL-6_1, IL-6_2,*  *TNF_1, TNF_2* | Hamilton Anxiety Rating Scale  (HAMA) and the Panic Disorder Severity Scale (PDSS) Chinese version. | Correlations  False discovery rate multiple comparison correction | (i) IL-4 DNAm higher in PD vs HC  (iia)The methylation levels of IL-4 DNAm  positively related to the severity of panic and anxiety symptoms. (iib)Hypermethylation of CSF2, CXCL8  and IL-4 associated with higher CA in PD |
|  |  |  |  |  |  |  |  |  |  |
| **(Misiak et al., 2015)** | N= 96  (48 FES; 48HC)  FES: 21/27  HC: 23/25  European (Poland) | Cross-sectional  Case-control | (ETISR-SF)  Composite  FEP+(CA)  FEP-(NonCA) | (i) Methylation of LINE-1 and BAGE between FES +, FE- vs HC  (ii) signature of CA on LINE and BAGE | Blood/Leucocytes  Bisulphite restriction assay  (BC + PCR) | Retrotransposon  BAGE/LINE | OPCRIT based on  DSM-IV and ICD-10 | (i) ANOVA  (ii) Linear regression  Age, gender and BMI, smoking, one-carbon metabolism parameters (Hcy, folate and vitamin B12); medication | (i) FES+ had lower LINE-1 methylation in comparison with FEP – or HC.  (ii) EA and total trauma predicted lower LINE-1 methylation in FES patients, |
| **EWAS studies** | | | | | | | | | |
| \| **(Weder et al., 2014)** \| N= 190 children  (94 CA + traumatized and 96 CA -)  79M/111F  17% European American, 38% Hispanic, 30%  African American, and 15% biracial \| Cross-sectional \| KSADS and CTQ and the PVI \| To identify novel DNAm markers associated with depression in maltreated children using the Illumina 450K BeadChip. \| Saliva  Genome-wide  Illumina 450K BeadChip array. \| Candidate genes were also explored for:  LC6A4, BDNF, NR3C1, FKBP5 \| Depression (MFQ)  Other psychiatric diagnoses (KSADS-PL; CBCL; CDC’ TRF; PTSD-CL) \| (I) LMM EWAS associated to depression  Demographic  variables age, sex, or race/ethnicity  (II) GEE to test the combined effect of CA and each CPG on depression  M-values (logit transformation in log2 scale) were used (heteroscedasticity) \| -ID3, GRIN1 and (TPPP) meth predicted depression.  -Independent effects of Meth and CA on depression (no interaction)  -CA_+ vs CA – differed in BDNF, NR3C1, FKBP5 \| \| --- \| --- \| --- \| --- \| --- \| --- \| --- \| --- \| --- \| --- \| \| **(Prados et al., 2015)** \| N=189  96 Borderline PD; 93 MDD  Borderline PD: 8M/88F MDD: 34M/59F  Swiss sample \| Cross-sectional \| CTQ \| Link between CA and EWAS DNAm in Borderline PD/MDD subjects \| Blood DNA (whole blood)  Genome-wide methylation was assessed using the Illumina 450 \| Hypothesis free \| MDD and Borderline PD diagnosis  (SCID-II and DIGS \| (1) LSVM model to test link EWAS meth on MDD/Borderline PD  (2) generated linear models. To test link between CTQ and EWAS meth  LAMBDA of L1-SVM algorithm to test sparseness \| CpGs within or near the following genes (*IL17RA, miR124-3, KCNQ2, EFNB1, OCA2, MFAP2, RPH3AL, WDR60, CST9L, EP400, A2ML1, NT5DC2, FAM163A* and *SPSB2*) differently methylated, either in Borderline PD compared with MDD or in relation to CA  Pathways:  inflammation  Neural excitability and development  or maintenance of nervous system  structure  of cell and regulation of cell functions  molecule  transport through cell membranes, calcium-ion-dependent  exocytosis in both endocrine and exocrine cells, cycle progression,  signal transduction, apoptosis and gene regulation  neural plasticity and neurogenesis. \| \| **(Mehta et al., 2013)** \| N:169  HC (CA +): 108  PTSD with SA: 32  PTSD non-SA: 29  HC(CA+): 30M/78F  PTSD SA+:5M/27F  PTSD Non-SA+:13M/6F  NA 93.5% African American \| Cross-sectional \| CTQ (abuse only)  TEI \| To interrogate DNAm in individuals with PTSD with or without childhood abuse vs HC with CA+ \| Blood (whole)  Genome wide analyses  Human Methylation  450k BeadChip \| Hypothesis free \| PTSD diagnosis where assessed using with the CAPS and the PSS scales \| General linear models  Sex, age, ethnicity, substance  abuse, and treatment \| Gene-expression and DNAm profiles were different in PTSD according to the exposure (or not) to childhood abuse.  These gene-expression changes were accompanied and likely mediated by changes in DNA methylation in the same loci to a much larger proportion in the childhood abuse (69%) vs. the non-child abuse-only group (34%) \| \| **(Uddin et al., 2010)** \| N=100  PTSD:23  Non-PTSD:77  (all at least with one PTE)  Overall sample: 40M/60F  14% white  79% African American  7% other race \| Cross-sectional \| Check list of 19 PTE \| (i) comparing uniquely methylated and unmethylated genes in PTSD versus non-PTSD and examining those overlapping across the two conditions \| Blood  Genome wide analysis  HM27 DNAm Analysis BeadChip by Illumina (27.000 CpG covering more than 14.000 genes \| Hypothesis free \| PCL-C \| McNemar’s χ2 test.  Checked that PTSS and non-PTSD participants did not differ in age, sex, race, or peripheral blood mononuclear cell count \| In PTSD participants there was a predominance of uniquely methylated genes involved in Inflammatory response (26 genes), immune response (38 genes) and innate immune response (11 genes) whereas there was a predominance of uniquely methylated genes involved in neurogenetic processes in non-affected PTSD (developmental process (50 genes) and generation of neurons (37 genes) \| \| **(Comes et al., 2020)** \| N=96 BD  50F/46M  100% of caucasians europeans  sample \| 1 year follow-up \| (CTS)  (non traumatic stress) \| (I) Exploring sites in vicinity to candidate genes previously implicated in stress response \| Peripheral blood  Genome wide/candidate gene  EpiTect  ® Bisulfite Kit from QIAGEN  (850.000 methylation sites) \| genes in vicinity to:  BDNF, FKBP5, IL6, SLC6A4, and OXTR \| Sample of Bipolar disorder parents (no instrument reported) \| Linear mixed effects model (LMM)  Cell composition, smoking, patients on medication were excluded. \| No locus withstood multiple correction testing  Limited evidence of association between DNAm and non-traumatic life events  Suggestive evidence of association of CTS with  *POU6F2*  *FER1L6* \| \| **(Arranz et al., 2021)** \| Sample 1  N= 49 Borderline PD CA+/47 Borderline PD CA-  and  HC=44  Sample 2  N = 293 Borderline PD patients and N = 114 controls  100% female and Caucasian ancestry \| Cross-sectional \| CTQ \| BORDERLINE PD with and without CA EWAS \| Peripheral blood  genome wide  (Illumina Infinium HumanMethylation450k beadchip) \| Hypothesis free \| SCID II  (DIB-R), McLean Screening  Instrument for Borderline Personality Disorder (MSIBorderline PD), \| Linear regressions  Age, PC,  and presence or absence of trauma in the first analysis,  and age and PC for the second and third analyses \| DNAm differences between Borderline PD+/Borderline PD- and controls  in  *POU5F1*  *GPR55*  *GGT6*  (REDOX)  *TNFRSF13C*  (CELL B ACTIV)  *FAM113B*  DISC2 (NS)  DNAm differences between  Borderline PD+/Borderline PD- and controls  No corrected significant p-value*: NKX2-1*  *PXDN*  *POU5F1*  Enrichment:  neurogenesis, regulation of cell differentiation and transport \| \| **(Lutz et al., 2017)**  **.** \| N:78  HC:26  MDD CA+:25  MDD CA-:27 \| Cross-sectional \| CECA \| (i) DNAm differences between MDD CA+ and HC and links with suicide and depression status \| Post-morten brains (Anterior cingulate cortex tissue)  Genome Wide  Illumina MiSeq (696,000 probes) \| Hypothesis free \| Diagnoses based on DSM-IV criteria. \| Linear regression analyses  Age, gender, and RNA integrity number \| (i) Top 3 genes different between MDD CA+ and HC are *LINGO3, POU3F1* and *ITGB1* involved in neurogenesis (myelinisation processes and oligodendrocytes formation) depression and suicide did not relate to such DNAm changes \| | | | | | | | | | |

DNA: Deoxyribonucleic acid; DNAm: DNA methylation CA: Childhood Adversity; EA: emotional abuse; SA: sexual abuse; CTS: childhood trauma screener; SCZ: Schizophrenia; FES: First Episode of Psychosis patients; HC: Healthy Controls; CTQ: Childhood Trauma Questionnaire; CTS: Conflict Tactics Scale/screener; MDD: major depressive disorder; BC: Bisulfite conversion; PIV: partner violence inventory; KSADS: Kiddie-Sads Child psychiatry Interview; ID3: DNA-Binding Protein Inhibitor ID–3; GRIN1: Glutamate Receptor, Ionotropic N-methyl-D-aspartate (NMDA) 1; TPP areP: Tubulin Polymerization Promoting Protein; GAD: generalized anxiety disorder; NR3C1: Glucocorticoiddd receptor gene; FKBP5: FK506 binding protein 5; BDNF: Brain Derived Neurotrophic factor; MAOA: Monoamine oxidase A gene; SKA2: Spindle And Kinetochore Associated Complex Subunit 2 gene; SLC6A4: Solute Carrier Family 6 Member 4 (serotonin transporter gene); HTR3A: 5-Hydroxytryptamine Receptor 3A gene; DNMT1: DNA Methyltransferase 1; OXTR: Oxytocin Receptor gene; GRIN2B Glutamate Ionotropic Receptor NMDA Type Subunit 2B; HPA: hypothalamic–pituitary–adrenal axis; CpG: Cytosine, phosphate Guanine (CpG island); SNP: single nucleotide polymorphism; CBCL: the child Behaviour Checklist; SCID: Structured Clinical Interview for the Diagnostic and Statistical Manual of Mental Disorders, Fourth Edition; PD: Panic Disorder;  Borderline PD: Borderline Personality Disorder; PTSD: post-traumatic stress disorder; IPV: Interpersonal violence; ADHD: *Attention deficit hyperactivity disorder;*DIGS: Diagnostic Interview for Genetic Studies; PART: A Population Based Study of Mental Health in the Stockholm County; EPD: Early parental death; MDI: the Major Depression; Inventory; LES: Life Experiences Survey; PSS: Perceived Stress Scale; HAM-A: Hamilton Anxiety Rating Scale; PMBCs: peripheral blood; DIB-R: Revised Diagnostic Interview for Borderline; EDE: Eating Disorder Examination; CTI: Childhood Trauma Interview; CAPTSDI:  Clinician-Administered Post- Traumatic Stress Disorder Scale; PPD: post-partum depression; PVI: Partner Violence Inventory; TLEQ: Traumatic Life-event questionnaire; SRIP: self- report inventory for PTSD; ETI: Early Trauma Inventory; PBMCs: in peripheral blood mononuclear cells; TEI: Trauma Event Inventory; CAPS: clinical administered PTSD Scale; mPSS: Modified PTSD Symptomatic Scale; TLS: Total Life Stress; BPSAQ: Brief Physical and Sexual Abuse Questionnaire;  ETISR-SF: Early Trauma Inventory Self Report-Short Form; LINE: Long interspersed nuclear elements; BAGE: B Melanoma Antigen; PCR: Polymerase chain reaction; OPCRIT: Operational Criteria Checklist for Psychotic Illness and Affective Illness; DSM-IV: Diagnostic and Statistical Manual of Mental Disorders (4^th^ edition); ICD-10: International Statistical Classification of Diseases and Related Health Problems; MACE: Maltreatment and Abuse Chronology of Exposure; CECA: Childhood Experience of Care and Abuse Interview; CCL3: C-C Motif Chemokine Ligand 3; CRP: C-Reactive Protein; CSF2: Colony Stimulating Factor 2; CXCL8: C-X-C Motif Chemokine Ligand 8; IFNG: Interferon Gamma; IL: Interleukin; TNF: Tumor necrosis factor; PHQ-9: Patient Health Questionnaire; MINI: Mini International Neuropsychiatric Interview; M-CIDI: Munich-Composite International Diagnostic Interview; BDI-II: Beck-Depression-Inventory-II; HSCL: Hopkins Symptoms Checklist-25; BSL-23: Borderline Symptoms Checklist-23; PANSS: Positive and Negative Syndrome Scale; MADRS: Montgomery–Asberg Depression Rating Scale; YMRS: Young Mania Rating Scale; GAF: Global Assessment of Functioning; RBANS: Repeatable Battery for the Assessment of Neuropsychological Status; BMI: Body Mass Index; CPZ: chlorpromazine; TPPP: Tubulin Polymerization Promoting Protein; PTE: Post Traumatic event; PCL-C: PTSD checklist; POU6F2: POU Class 6 Homeobox 2; FER1L6: Fer-1 Like Family Member 6; SCID II: Structured clinical interview for DSM IV axis II personality disorders; NKX2-1: NK2 Homeobox 1; PXDN: Peroxidasin; POU5F1: POU Class 5 Homeobox 1; GPR55: G Protein-Coupled Receptor 55; GGT6: Gamma-Glutamyltransferase 6; *KCNQ2:* Potassium Voltage-Gated Channel Subfamily Q Member 2; *EFNB1:* Ephrin-B1

*; OCA2:* the P gene; *MFAP2:* Microfibril Associated Protein 2; *RPH3AL:* Rabphilin 3A Like Without C2 Domains*; WDR60 (*dynein 2 intermediate chain 1; *CST9L:* Cystatin 9 Like; *EP400:* E1A; Binding Protein P400; *A2ML1:* Alpha-2-Macroglobulin Like 1; *NT5DC2:* 5'-Nucleotidase Domain Containing 2; *FAM163A:* Family With Sequence Similarity 163 Member A; *SPSB2*: SplA/Ryanodine Receptor Domain And SOCS Box Containing 2; TNFRSF13C: TNF Receptor Superfamily Member 13C; FAM113B: Family with sequence similarity 113, member B, isoform CRA a; DISC2: a noncoding RNA molecule antisense to DISC1 (disruoted in Schizophrenia); LINGO3: Leucine Rich Repeat And Ig Domain Containing 3; POU3F1: POU Class 3 Homeobox 1; ITGB1: Integrin Subunit Beta 1; LMM: Linear mixed models; GEE: generalized estimating equation; LSVM: linear Support Vector Machine; HM27: Humanmethylation27. HAMD: Hamilton Depression Rating Scale; HAMA: Hamilton Anxiety Rating Scale; SOFAS: Social and Occupational Functioning Assessment Scale; FU: follow-up; SCID: The Structured Clinical Interview for DSM-IV Axis I Disorders.

**SUPLEMENTARY METHODS**

**Search Strategy and Selection Criteria**

The main electronic searches were conducted on MEDLINE, EMBASE, and PsycINFO, through Ovid provider, up to May 2019, and were updated in April 2020. Relevant papers that were published after that date were also considered until the date of the submission in June 2021.

For articles included in sections 2 (“*Evidence of epigenetic processes in major transdiagnostic pathways”)* We searched Medical Subject Headings and keywords related to: (1)

Epigenetics (including Epigenetics, EWAS,”epigenomics”,CpG island, DNA Methylation); (2) Mental health (covering the different diagnosis and conditions of interest);

using the Boolean operator “AND” (full list of search terms provided below).

For papers included in section 3 *(“The epigenetic signature of childhood adversity and cannabis use”),* two different searches were conducted:

For the search related to childhood adversity we searched Medical Subject Headings and keywords related to: (1) childhood adversity (including various adversities such as abuse, neglect and other life events etc): (2)

Epigenetics (including Epigenetics, EWAS,”epigenomics”,CpG island, DNA Methylation); (3) Mental health (covering the different terms such as mental condition or psychopathy) were combined using the Boolean operator “AND”.

For the search related to cannabis use we searched Medical Subject Headings and keywords related to: (1) cannabis use ( with term ssuch as Cannabis or “cannabis use”) (2)

Epigenetics (including Epigenetics, EWAS,”epigenomics”,CpG island, DNA Methylation); (3) Mental health (covering the different terms such as mental condition or psychiatry) were combined using the Boolean operator “AND” (full list of search terms for both searches are provided below).

Titles and abstracts, and subsequently selected eligible full texts of articles were screened independently by one author (LA) and checked by a second author (GT). Titles and abstract discrepancies were resolved through discussion at a project group meeting. A cross-reference search extracted titles/abstracts from identified reviews, and a full-text check of potentially eligible studies from these reviews was conducted by one author (L.A). When data extraction was conducted and new potentially eligible studies were detected from included studies, these were also considered for inclusion. An OVID alert was created for each of the searches so that the main author (L.A) could add new potentially eligible papers until the date of the submission.

*Inclusion and exclusion criteria*

*The inclusion criteria differed depending on the section of the review.*

Section 2 “Evidence of epigenetic processes in major transdiagnostic pathways’

In this section we focused on articles that examined an association between epigenetic modifications in the form of DNA Methylation (DNAm), in humans, and one of the conditions of interest (namely Eating disorders (anorexia nervosa and bulimia), Post traumatic stress disorder (PTSD), Anxiety disorders, Psychotic disorders (including Bipolar Disorder), Depression, Autism) or a specific psychopathological measure (for example depressive symptoms in the general population). We restricted our inclusion to publications that were conducted in candidate genes and Epigenome Wide Association Studies (EWAS). The goal was not to provide a systematic summary of all the papers fulfilling these criteria but to select the papers that were the most relevant and representative of each finding: for example, if 5 papers were found for one same gene, the ones judged to be most relevant were selected and reported. After conducting a first screening of the eligible papers, the most common represented biological pathways were delineated, namely the serotoninergic, dopaminergic pathways, dysfunction related to the excitatory and inhibitory balance (including the glutamatergic and GABAergic function), synaptic plasticity and neurogenesis, the immune system and inflammation et oxidative stress. Biological systems that fell outside these ones were finally not considered, given word limit constraints and to limit the scope of the review. For example, there were new potential pathways that are emerging as important for epigenetics in mental health such as mitochondrial dysfunction. However, given that evidence linking a dysfunction in this pathway is involved in major mental conditions, we decided to leave it outside of this work in order to make the review more comprehensive and compact.

Section 3 “The epigenetic signature of childhood adversity and cannabis use”

*Section on childhood adversity*

We included publications that examined a link between childhood adversity (also including papers looking at more recent life events) and epigenetic changes in samples of patients with the aforementioned mental condition; or that linked childhood adversity with DNAm and any clinical outcome in such samples; as for the previous sections we focused on candidate genes studies and EWAS and restricted to human studies.

*Section on cannabis use*

We included publications that examined a link between childhood adversity and epigenetic changes in samples of patients with the aforementioned mental condition; or that linked childhood adversity with DNAm and any clinical outcome in such samples. However, and against our initial prediction we found that studies in clinical samples were lacking. Therefore, in this section we decided to also include papers that looked at the association between cannabis use and DNAm (candidate genes and EWAS) in the general population.

*Exclusion criteria*

We did not focus on studies in animal models, studies that were not published in English, French, Spanish and Italian.

**Data extraction methodology**

Section 2 “Evidence of epigenetic processes in major transdiagnostic pathways”

For each reported association between DNAm and a clinical outcome or a condition of interest we reported, narratively the text whether there was a dysregulation, a reduced/increased (or hyper/hypo) DNAm, or a enrichment of a specific pathway. We reported, for each study, whether it was a candidate gene study or an EWAS, the tissue used, in which condition and to which pathway of interest that gene belong and the association of DNAm with a particular clinical or biological outcome. This information is also gathered in Table 3 which provide a summary of the above. In some occasions in the text, we refer to table 3 rather than reporting all the details. Table 1 summarised the names and basic function of each gene, allowing the reader to have a summary of the relevant genes mentioned in the review (rather than defining each gene in the text directly).

Section 3 **The epigenetic signature of childhood adversity and cannabis use**

*Section on childhood adversity*

In addition to reporting findings narratively as in section 2, Table 2 provides a summary of each of the relevant studies included. The table is separated by a Candidate genes study, and an EWAS section. The candidate genes section is divided into studies examining genes involved in the Hypothalamic Pituitary Adrenal Axis, the oxytocin pathway, the serotoninergic pathway, neurogenesis, and other pathway of interest. The selection of these pathways was made based on the observation that they were the most explored and relevant systems in the context of childhood adversity and DNAm. However, and contrary to section 2, here we also mentioned papers that explored more novel or preliminary pathways (described in the “other pathway of interest” section) The table extracts for each study the name of the author, demographic characteristics such as sample size and gender and clinical population, design, type of instrument used to assess childhood adversity, the goal of the analyses of interest, the DNAm collection methods, the studied gene, how the clinical information was assessed, statistic methods (including covariates included), and the main findings (trying to report when available the direction of the association between childhood adversity and DNAm and with the clinical outcome or condition). Data on section 3 is also gathered in Table 3 similarly than described above.

Section on cannabis use

Data relative to this section was reported narratively, in the same way than for section 2 and 3 but without a detailed table 2. As mentioned above, given the low number of studies in clinical samples, findings related to the general population were considered.

Figures 1 and 2 are illustration on the main findings presented in the review, combining sections 2 and 3 together.

Figure 1 summarises the evidence presented in this review. It illustrates :1) (green and blue rows) the suggested links between childhood adversity (CA), and Cannabis use (CU) and DNAm changes mapping to biological pathways which are also functionally related (Serotoninergic, Dopaminergic pathways, Glutamatergic & GABAergic pathway, Neurogenesis, Immune system & Inflammation and Oxidative stress).
2) The blue and beige rows illustrate the evidence, from case control studies, of an association between DNAm changes in these pathways and the major mental health conditions (Eating Disorders (anorexia nervosa and bulimia nervosa, Post-traumatic stress disorder, Anxiety Disorders, Psychotic Disorder, Bipolar disorders, Depression and Autism); 3) the potential mediating role of the DNAm changes mapping to these between exposure to CA and CU and risk to develop mental health conditions.
The thickness of the lines shows the robustness of the evidence reported in the literature review. The items “genotype: and “other risk factors” are added to highlight the influence of genetic factors and environmental counfounders in DNAm studies

Figure 2 illustrates the evidence from candidate gene studies linking childhood adversity (CA) with DNAm in CpG sites located in the genes NR3C1, FKBP5, SKA2 and CA, with various conditions and various clinical outcomes.
In columns blue and beige, CA+ (with an arrow pointing up) reflects the presence of a positive association between the DNAm in probes located in those genes and CA; CA- (with an arrow pointing down) reflects a negative association. The beige column shows the disorder in which that association has been found. Lastly, the green column shows the presence of evidence linking DNAm, with a particular clinical phenotype; CA+ indicated that that the association between DNAm and the clinical outcome was related to CA.

|  |
| --- |

1. exp epigenetics/ or exp DNA methylation/

2. EWAS.mp.

3. epigenomics.mp.

4. exp CpG island/

5. exp DNA Methylation/ or exp Epigenomics/ or exp CpG Islands/

6. exp EPIGENETICS/

7. DNA methylation.mp.

8. CpG islands.mp.

9. 1 or 4 or 5 or 6

10. 2 or 3 or 7 or 8

11. exp ALCOHOLISM/

12. Alcohol.mp.

13. exp alcohol/ or exp alcoholism/ or exp alcohol abuse/

14. exp ALCOHOLISM/

15. exp Alcohol Abuse/

16. alcohol dependance.mp.

17. exp Alzheimer Disease/

18. 14 or 15

19. exp Alzheimer disease/

20. exp Alzheimer's Disease/

21. 17 or 19 or 20

22. exp psychosis/

23. exp schizophrenia/

24. exp psychosis/

25. exp schizophrenia/

26. 22 or 23

27. 24 or 25

28. exp PSYCHOSIS/

29. exp SCHIZOPHRENIA/

30. psychos*.mp.

31. Schizophren*.mp.

32. 28 or 29

33. exp Bipolar Disorder/

34. exp bipolar disorder/

35. exp Bipolar Disorder/

36. 33 or 34 or 35

37. exp Autism Spectrum Disorder/ or exp Autistic Disorder/

38. exp autism/

39. autistic disorder.mp.

40. autism.mp.

41. exp Autism Spectrum Disorders/

42. 37 or 38 or 41

43. exp DEPRESSION/

44. exp Depressive Disorder/

45. 43 or 44

46. exp depression/

47. exp Major Depression/

48. 45 or 46 or 47

49. depression.mp.

50. exp SMOKING/ or exp TOBACCO SMOKING/

51. exp tobacco/ or exp smoking/ or exp tobacco dependence/

52. exp TOBACCO SMOKING/

53. 50 or 51 or 52

54. smoking tobacco.mp.

55. psychotropic medication.mp.

56. exp antidepressant agent/ or exp psychotropic agent/ or exp neuroleptic agent/ or exp anxiolytic agent/

57. exp Antidepressant Drugs/ or exp Drug Therapy/ or exp Neuroleptic Drugs/

58. exp Antipsychotic Agents/ or exp Psychotropic Drugs/

59. 9 or 10

60. 11 or 13 or 18

61. Alzheimer.mp.

62. bipolar disorder.mp.

63. 12 or 16 or 30 or 31 or 39 or 40 or 49 or 54 or 55 or 61 or 62

64. 26 or 27 or 32

65. 56 or 57 or 58

66. 21 or 36 or 42 or 48 or 53 or 60 or 64 or 65

67. 63 or 66

68. exp psychiatry/

69. exp PSYCHIATRY/

70. psychiatry.mp.

71. exp PSYCHIATRY/

72. 68 or 69 or 70 or 71

73. 59 and 67 and 72

Full searches conducted via OVID provided in sections 3 (“**The epigenetic signature of childhood adversity and cannabis use**”**)**

For childhood adversity the full search was:

1. Stress, Psychological/

2. Physical Abuse/

3. sex offenses/ or child abuse, sexual/ or rape/

4. emotional abuse.mp.

5. emotional neglect.mp.

6. Physical neglect.mp.

7. Bullying/

8. separation from parents.mp.

9. being taken into care.mp.

10. interpersonal trauma.mp.

11. traumatic exp*.mp.

12. trauma exp*.mp.

13. Early life event*.mp.

14. early life stress.mp.

15. 1 or 2 or 3 or 4 or 5 or 6 or 7 or 8 or 9 or 10 or 11 or 12 or 13 or 14

16. stress/ or exp acute stress/ or exp chronic stress/ or exp early life stress/ or exp emotional stress/ or exp interpersonal stress/ or exp life stress/ or exp maternal stress/ or exp mental stress/ or exp parental stress/ or exp school stress/

17. child abuse/ or exp abuse/ or exp domestic violence/ or exp child neglect/ or exp child sexual abuse/

18. exp neglect/ or exp child neglect/

19. exp physical abuse/

20. exp emotional abuse/

21. exp sexual bullying/ or exp bullying/

22. 15 or 16 or 17 or 18 or 19 or 20

23. stress/ or exp chronic stress/ or exp environmental stress/ or exp psychological stress/

24. exp CHILD ABUSE/ or exp EMOTIONAL ABUSE/ or exp PHYSICAL ABUSE/ or exp SEXUAL ABUSE/

25. exp CHILD NEGLECT/

26. exp BULLYING/

27. trauma/ or exp emotional trauma/

28. 22 or 23 or 24 or 25 or 26 or 27

29. exp Epigenomics/

30. EWAS.mp.

31. epigen*.mp.

32. exp CpG Islands/

33. exp Methylation/ or exp DNA Methylation/

34. 29 or 30 or 31 or 32 or 33

35. exp epigenetics/ or exp epigenesis/ or exp DNA methylation/

36. exp CpG island/

37. 34 or 35 or 36

38. exp EPIGENETICS/

39. 37 or 38

40. exp Mental Disorders/

41. Psychiatric vulnerability.mp.

42. mental diseases.mp.

43. 40 or 41 or 42

44. psychopathology/ or adolescent psychopathology/ or child psychopathology/ or psychiatric symptoms/

45. 43 or 44

46. psychopathology.mp. or mental disease/

47. 45 or 46

48. 28 and 39 and 47

49. 29 or 30 or 31 or 32 or 36 or 38

50. exp epigenetics/

51. exp epigenesis/

52. 29 or 30 or 31 or 36 or 50 or 51

53. 40 or 41 or 42

54. 28 and 52 and 53

55. 1 or 2 or 3 or 4 or 5 or 6 or 7 or 8 or 9 or 10 or 11 or 12 or 13 or 14 or 16 or 17 or 18 or 19 or 20 or 21 or 23 or 24 or 25 or 26 or 27

56. Epigenesis, Genetic/

57. 29 or 30 or 31 or 32 or 38 or 56

58. 53 and 55 and 57

59. mental disease/ or exp addiction/ or exp anxiety disorder/ or exp dissociative disorder/ or exp mood disorder/ or exp neurosis/ or exp personality disorder/ or exp psychosis/ or exp psychosomatic disorder/ or exp psychotrauma/

60. exp mental disorders/ or exp anxiety disorders/ or exp dissociative disorders/ or exp mood disorders/ or neurotic disorders/ or exp paraphilic disorders/ or personality disorders/ or exp "schizophrenia spectrum and other psychotic disorders"/ or exp sexual dysfunctions, psychological/ or exp somatoform disorders/ or exp substance-related disorders/ or exp "trauma and stressor related disorders"/

61. mental disorders/ or exp affective disorders/ or exp anxiety disorders/ or exp dissociative disorders/ or exp neurosis/ or exp personality disorders/ or exp psychosis/ or exp schizoaffective disorder/

62. 41 or 59 or 60 or 61

63. 34 and 55 and 62

64. 2 or 3 or 4 or 5 or 6 or 7 or 8 or 10 or 11 or 12 or 14 or 17 or 18 or 19 or 20 or 21 or 24 or 25 or 26 or 27

65. 34 and 62 and 64

For cannabis use the full search was:

1. exp epigenetics/

2. exp DNA methylation/

3. exp psychiatry/

4. exp mental health/

5. exp cannabis/

6. exp Epigenomics/

7. exp DNA Methylation/

8. exp Psychiatry/

9. exp Mental Health/

10. exp Cannabis/

11. exp Epigenetics/

12. exp Psychiatry/

13. exp Mental Health/

14. exp Cannabis/

15. epigen*.mp.

16. methylation.mp.

17. "cannabis use".mp.

18. psychiatry.mp.

19. 5 or 10 or 14 or 17

20. 1 or 2 or 6 or 7 or 11 or 15 or 16

21. 3 or 4 or 8 or 9 or 12 or 13 or 18

22. 19 and 20 and 21

23. from 22 keep 1-22

24. 19 and 20

**REFERENCES**

Arranz, M. J., Gallego-Fabrega, C., Martín-Blanco, A., Soler, J., Elices, M., Dominguez-Clavé, E., . . . Pascual, J. C. (2021). A genome-wide methylation study reveals X chromosome and childhood trauma methylation alterations associated with borderline personality disorder. *Translational Psychiatry, 11*(1), 5. doi:10.1038/s41398-020-01139-z

Boks, M. P., van Mierlo, H. C., Rutten, B. P., Radstake, T. R., De Witte, L., Geuze, E., . . . Broen, J. C. (2015). Longitudinal changes of telomere length and epigenetic age related to traumatic stress and post-traumatic stress disorder. *Psychoneuroendocrinology, 51*, 506-512.

Bustamante, A. C., Aiello, A. E., Galea, S., Ratanatharathorn, A., Noronha, C., Wildman, D. E., & Uddin, M. (2016). Glucocorticoid receptor DNA methylation, childhood maltreatment and major depression. *Journal of Affective Disorders, 206*(Supplement C), 181-188. doi:<https://doi.org/10.1016/j.jad.2016.07.038>

Bustamante, A. C., Aiello, A. E., Guffanti, G., Galea, S., Wildman, D. E., & Uddin, M. (2018). FKBP5 DNA methylation does not mediate the association between childhood maltreatment and depression symptom severity in the Detroit Neighborhood Health Study. *Journal of Psychiatric Research, 96*(Supplement C), 39-48. doi:<https://doi.org/10.1016/j.jpsychires.2017.09.016>

Comes, A. L., Czamara, D., Adorjan, K., Anderson-Schmidt, H., Andlauer, T. F. M., Budde, M., . . . Heilbronner, U. (2020). The role of environmental stress and DNA methylation in the longitudinal course of bipolar disorder. *International Journal of Bipolar Disorder, 8*(1), 9. doi:10.1186/s40345-019-0176-6

Domschke, K., Tidow, N., Kuithan, H., Schwarte, K., Klauke, B., Ambrée, O., . . . Kersting, A. (2012). Monoamine oxidase A gene DNA hypomethylation–a risk factor for panic disorder? *International Journal of Neuropsychopharmacology, 15*(9), 1217-1228.

Domschke, K., Tidow, N., Schrempf, M., Schwarte, K., Klauke, B., Reif, A., . . . Deckert, J. (2013). Epigenetic signature of panic disorder: a role of glutamate decarboxylase 1 (GAD1) DNA hypomethylation? *Progress in Neuro-Psychopharmacology and Biological Psychiatry, 46*, 189-196.

Engdahl, E., Alavian-Ghavanini, A., Forsell, Y., Lavebratt, C., & Rüegg, J. (2021). Childhood adversity increases methylation in the GRIN2B gene. *J Psychiatry Research, 132*, 38-43. doi:10.1016/j.jpsychires.2020.09.022

Farrell, C., Doolin, K., O’Leary, N., Jairaj, C., Roddy, D., Tozzi, L., . . . Nemoda, Z. (2018). DNA methylation differences at the glucocorticoid receptor gene in depression are related to functional alterations in hypothalamic–pituitary–adrenal axis activity and to early life emotional abuse. *Psychiatry research, 265*, 341-348.

Kaminsky, Z., Wilcox, H., Eaton, W., Van Eck, K., Kilaru, V., Jovanovic, T., . . . Ressler, K. (2015). Epigenetic and genetic variation at SKA2 predict suicidal behavior and post-traumatic stress disorder. *Translational psychiatry, 5*(8), e627.

Kang, H.-J., Kim, J.-M., Stewart, R., Kim, S.-Y., Bae, K.-Y., Kim, S.-W., . . . Yoon, J.-S. (2013). Association of SLC6A4 methylation with early adversity, characteristics and outcomes in depression. *Progress in Neuro-Psychopharmacology and Biological Psychiatry, 44*, 23-28.

Kimmel, M., Clive, M., Gispen, F., Guintivano, J., Brown, T., Cox, O., . . . Kaminsky, Z. (2016). Oxytocin receptor DNA methylation in postpartum depression. *Psychoneuroendocrinology, 69*, 150-160. doi:10.1016/j.psyneuen.2016.04.008

Klengel, T., Mehta, D., Anacker, C., Rex-Haffner, M., Pruessner, J. C., & Pariante, C. M. (2013). Allele-specific FKBP5 DNA demethylation mediates gene-childhood trauma interactions. *Nature Neuroscience, 16*. doi:10.1038/nn.3275

Klinger-König, J., Hertel, J., Van der Auwera, S., Frenzel, S., Pfeiffer, L., Waldenberger, M., . . . Homuth, G. (2019). Methylation of the FKBP5 gene in association with FKBP5 genotypes, childhood maltreatment and depression. *Neuropsychopharmacology, 44*(5), 930-938.

Koenen, K. C., Uddin, M., Chang, S. C., Aiello, A. E., Wildman, D. E., Goldmann, E., & Galea, S. (2011). SLC6A4 methylation modifies the effect of the number of traumatic events on risk for posttraumatic stress disorder. *Depression and anxiety, 28*(8), 639-647.

Labonte, B., Azoulay, N., Yerko, V., Turecki, G., & Brunet, A. (2014). Epigenetic modulation of glucocorticoid receptors in posttraumatic stress disorder. *Translational psychiatry, 4*(3), e368.

Lutz, P.-E., Tanti, A., Gasecka, A., Barnett-Burns, S., Kim, J. J., Zhou, Y., . . . Almeida, D. (2017). Association of a history of child abuse with impaired myelination in the anterior cingulate cortex: convergent epigenetic, transcriptional, and morphological evidence. *American Journal of Psychiatry*, appi. ajp. 2017.16111286.

Martin-Blanco, A., Ferrer, M., Soler, J., Salazar, J., Vega, D., & Andion, O. (2014). Association between methylation of the glucocorticoid receptor gene, childhood maltreatment, and clinical severity in borderline personality disorder. *Journal of Psychiatry Research, 57*. doi:10.1016/j.jpsychires.2014.06.011

Mehta, D., Klengel, T., Conneely, K. N., Smith, A. K., Altmann, A., Pace, T. W., . . . Mercer, K. B. (2013). Childhood maltreatment is associated with distinct genomic and epigenetic profiles in posttraumatic stress disorder. *Proceedings of the national academy of sciences, 110*(20), 8302-8307.

Melas, P. A., Wei, Y., Wong, C. C., Sjöholm, L. K., Åberg, E., Mill, J., . . . Lavebratt, C. (2013). Genetic and epigenetic associations of MAOA and NR3C1 with depression and childhood adversities. *International Journal of Neuropsychopharmacology, 16*(7), 1513-1528.

Misiak, B., Karpiński, P., Szmida, E., Grąźlewski, T., Jabłoński, M., Cyranka, K., . . . Frydecka, D. (2020). Adverse Childhood Experiences and Methylation of the FKBP5 Gene in Patients with Psychotic Disorders. *Journal of Clinical Medicine, 9*(12). doi:10.3390/jcm9123792

Misiak, B., Szmida, E., Karpinski, P., Loska, O., Sasiadek, M. M., & Frydecka, D. (2015). Lower LINE-1 methylation in first-episode schizophrenia patients with the history of childhood trauma. *Epigenomics, 7*(8), 1275-1285. doi:10.2217/epi.15.68

Moser, D. A., Paoloni-Giacobino, A., Stenz, L., Adouan, W., Manini, A., Suardi, F., . . . Rusconi-Serpa, S. (2015). BDNF methylation and maternal brain activity in a violence-related sample. *PLoS One, 10*(12), e0143427.

Peng, H., Zhu, Y., Strachan, E., Fowler, E., Bacus, T., Roy-Byrne, P., . . . Zhao, J. (2018). Childhood Trauma, DNA Methylation of Stress-related Genes, and Depression: Findings from Two Monozygotic Twin Studies. *Psychosomatic Medicine*. doi:10.1097/psy.0000000000000604

Perroud, N., Paoloni-Giacobino, A., Prada, P., Olie, E., Salzmann, A., & Nicastro, R. (2011). Increased methylation of glucocorticoid receptor gene (NR3C1) in adults with a history of childhood maltreatment: a link with the severity and type of trauma. *Translational Psychiatry, 1*. doi:10.1038/tp.2011.60

Perroud, N., Zewdie, S., Stenz, L., Adouan, W., Bavamian, S., & Prada, P. (2016). Methylation of serotonin receptor 3a in Adhd, borderline personality, and bipolar disorders: link with severity of the disorders and childhood maltreatment. *Depression and anxiety, 33*. doi:10.1002/da.22406

Prados, J., Stenz, L., Courtet, P., Prada, P., Nicastro, R., & Adouan, W. (2015). Borderline personality disorder and childhood maltreatment: a genome-wide methylation analysis. *Genes Brain Behavior, 14*. doi:10.1111/gbb.12197

Radtke, K., Schauer, M., Gunter, H. M., Ruf-Leuschner, M., Sill, J., Meyer, A., & Elbert, T. (2015). Epigenetic modifications of the glucocorticoid receptor gene are associated with the vulnerability to psychopathology in childhood maltreatment. *Translational psychiatry, 5*(5), e571.

Sadeh, N., Spielberg, J. M., Logue, M. W., Wolf, E. J., Smith, A. K., Lusk, J., . . . McGlinchey, R. E. (2016). SKA2 methylation is associated with decreased prefrontal cortical thickness and greater PTSD severity among trauma-exposed veterans. *Molecular psychiatry, 21*(3), 357-363.

Sadeh, N., Wolf, E. J., Logue, M. W., Hayes, J. P., Stone, A., Griffin, L. M., . . . Miller, M. W. (2016). Epigenetic variation at SKA2 predicts suicide phenotypes and internalizing psychopathology. *Depression and anxiety, 33*(4), 308-315.

Schechter, D. S., Moser, D. A., Paoloni-Giacobino, A., Stenz, L., Gex-Fabry, M., Aue, T., . . . Manini, A. (2015). Methylation of NR3C1 is related to maternal PTSD, parenting stress and maternal medial prefrontal cortical activity in response to child separation among mothers with histories of violence exposure. *Frontiers in psychology, 6*.

Schechter, D. S., Moser, D. A., Pointet, V. C., Aue, T., Stenz, L., Paoloni-Giacobino, A., . . . Dayer, A. G. (2017). The association of serotonin receptor 3A methylation with maternal violence exposure, neural activity, and child aggression. *Behavioural Brain Research, 325*(Part B), 268-277. doi:<https://doi.org/10.1016/j.bbr.2016.10.009>

Simons, R. L., Lei, M. K., Beach, S. R. H., Cutrona, C. E., & Philibert, R. A. (2017). Methylation of the oxytocin receptor gene mediates the effect of adversity on negative schemas and depression. *Dev Psychopathol, 29*(3), 725-736. doi:10.1017/s0954579416000420

Sipahi, L., Wildman, D. E., Aiello, A. E., Koenen, K. C., Galea, S., Abbas, A., & Uddin, M. (2014). Longitudinal epigenetic variation of DNA methyltransferase genes is associated with vulnerability to post-traumatic stress disorder. *Psychological medicine, 44*(15), 3165-3179.

Smearman, E. L., Almli, L. M., Conneely, K. N., Brody, G. H., Sales, J. M., Bradley, B., . . . Smith, A. K. (2016). Oxytocin receptor genetic and epigenetic variations: association with child abuse and adult psychiatric symptoms. *Child development, 87*(1), 122-134.

Steiger, H., Labonté, B., Groleau, P., Turecki, G., & Israel, M. (2013). Methylation of the glucocorticoid receptor gene promoter in bulimic women: associations with borderline personality disorder, suicidality, and exposure to childhood abuse. *International Journal of Eating Disorders, 46*(3), 246-255.

Thaler, L., Gauvin, L., Joober, R., Groleau, P., de Guzman, R., Ambalavanan, A., . . . Steiger, H. (2014). Methylation of BDNF in women with bulimic eating syndromes: Associations with childhood abuse and borderline personality disorder. *Progress in Neuro-Psychopharmacology and Biological Psychiatry, 54*(Supplement C), 43-49. doi:<https://doi.org/10.1016/j.pnpbp.2014.04.010>

Tozzi, L., Farrell, C., Booij, L., Doolin, K., Nemoda, Z., Szyf, M., . . . Frodl, T. (2017). Epigenetic Changes of FKBP5 as a Link Connecting Genetic and Environmental Risk Factors with Structural and Functional Brain Changes in Major Depression. *Neuropsychopharmacology*. doi:10.1038/npp.2017.290

Tyrka, A., Parade, S., Welch, E., Ridout, K., Price, L., Marsit, C., . . . Carpenter, L. (2016). Methylation of the leukocyte glucocorticoid receptor gene promoter in adults: associations with early adversity and depressive, anxiety and substance-use disorders. *Translational psychiatry, 6*(7), e848.

Uddin, M., Aiello, A. E., Wildman, D. E., Koenen, K. C., Pawelec, G., de Los Santos, R., . . . Galea, S. (2010). Epigenetic and immune function profiles associated with posttraumatic stress disorder. *Proceedings of the national academy of sciences, 107*(20), 9470-9475.

Wang, W., Feng, J., Ji, C., Mu, X., Ma, Q., Fan, Y., . . . Zhu, F. (2017). Increased methylation of glucocorticoid receptor gene promoter 1F in peripheral blood of patients with generalized anxiety disorder. *Journal of Psychiatric Research, 91*(Supplement C), 18-25. doi:<https://doi.org/10.1016/j.jpsychires.2017.01.019>

Weder, N., Zhang, H., Jensen, K., Yang, B. Z., Simen, A., Jackowski, A., . . . Perepletchikova, F. (2014). Child abuse, depression, and methylation in genes involved with stress, neural plasticity, and brain circuitry. *Journal of the American Academy of Child & Adolescent Psychiatry, 53*(4), 417-424. e415.

Wolf, E. J., Logue, M. W., Stoop, T. B., Schichman, S. A., Stone, A., Sadeh, N., . . . Miller, M. W. (2017). Accelerated DNA Methylation Age: Associations with PTSD and Mortality. *Psychosomatic Medicine*.

Yehuda, R., Daskalakis, N. P., Bierer, L. M., Bader, H. N., Klengel, T., Holsboer, F., & Binder, E. B. (2016). Holocaust Exposure Induced Intergenerational Effects on FKBP5 Methylation. *Biol Psychiatry, 80*(5), 372-380. doi:10.1016/j.biopsych.2015.08.005

Yehuda, R., Flory, J. D., Bierer, L. M., Henn-Haase, C., Lehrner, A., Desarnaud, F., . . . Meaney, M. J. (2015). Lower methylation of glucocorticoid receptor gene promoter 1 F in peripheral blood of veterans with posttraumatic stress disorder. *Biological Psychiatry, 77*(4), 356-364.

Zou, Z., Huang, Y., Wang, J., Min, W., & Zhou, B. (2020). DNA methylation of IL-4 gene and the association with childhood trauma in panic disorder. *Psychiatry Res, 293*, 113385. doi:10.1016/j.psychres.2020.113385
